# Supplementary figures and images for: A reliance on human habitats is key to the success of an introduced predatory reptile
Source: PLoS One. 2025 Feb 5;20(2):e0310352. doi: 10.1371/journal.pone.0310352 (PMC11798526; doi:10.1371/journal.pone.0310352)

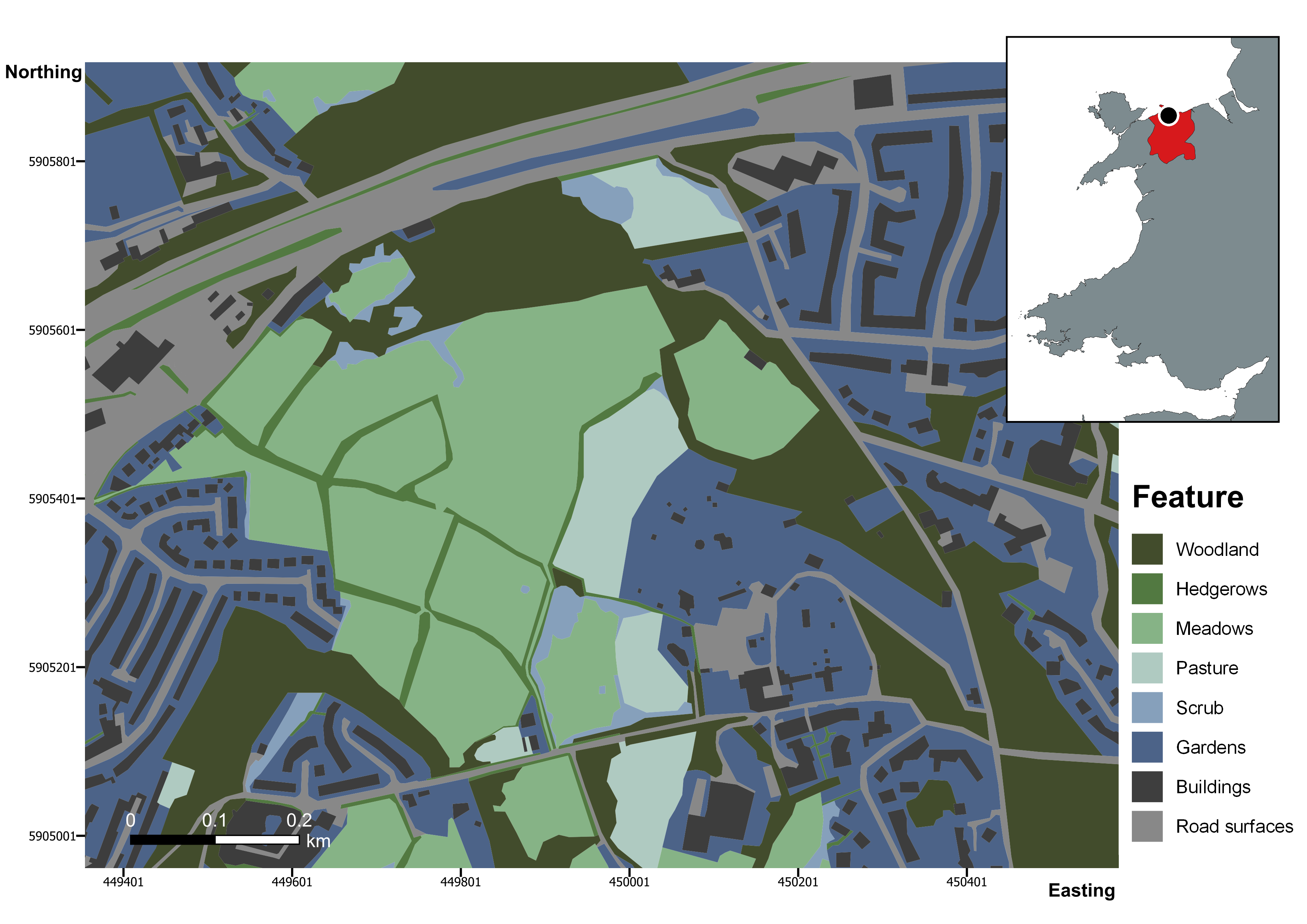

Supplement: S1 Fig — Inset map shows location of the study site in Wales, UK, with Conwy County highlighted red. Map created using QGIS [64]. (TIF) [file pone.0310352.s001.tif]

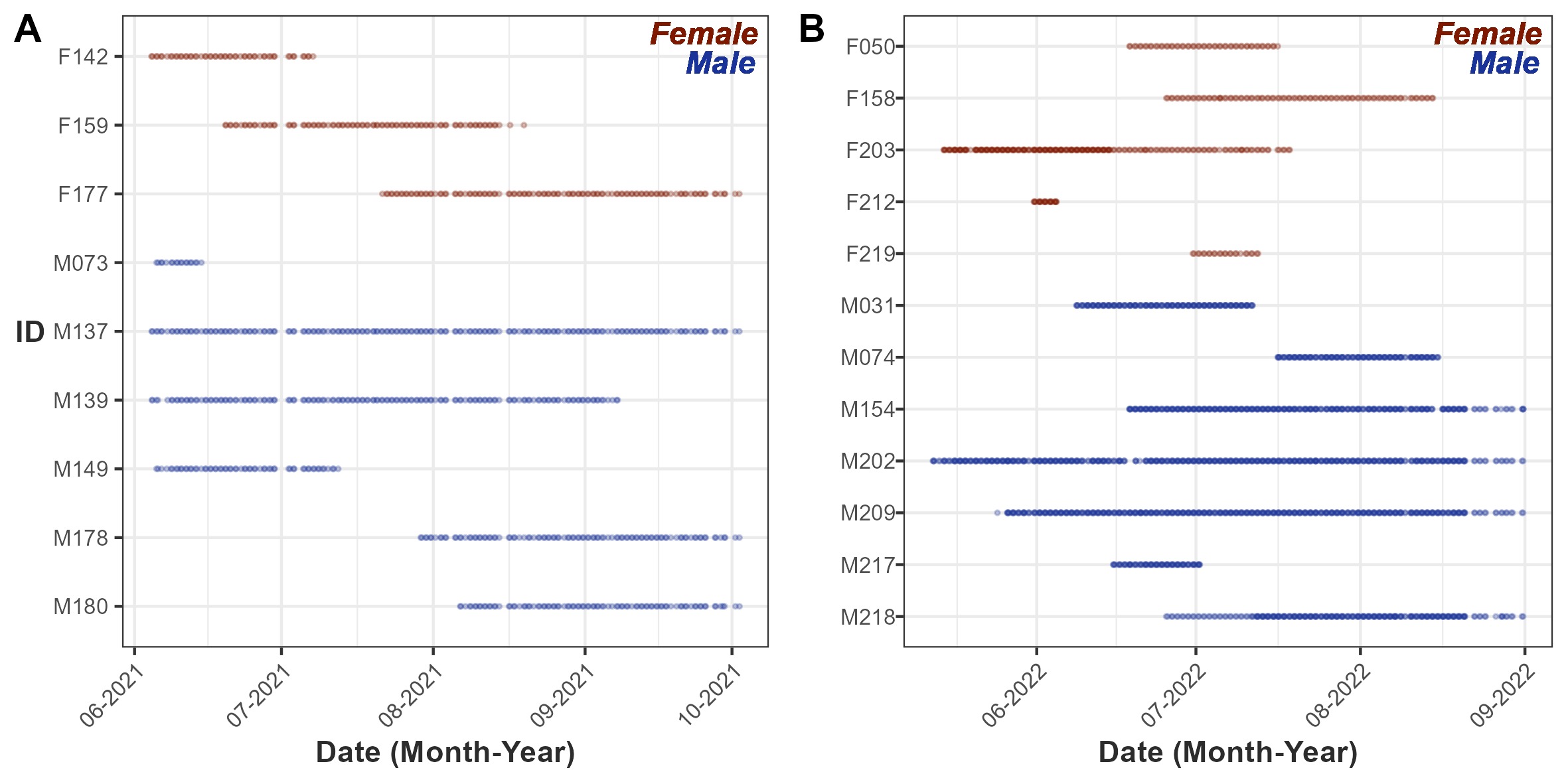

Supplement: S2 Fig — Tracking periods for 21 tracked snakes over (A) 2021 and (B) 2022. Each point represents a tracking occasion where the snake was located, with a higher density of points representing increased tracking frequency. (TIF) [file pone.0310352.s002.tif]

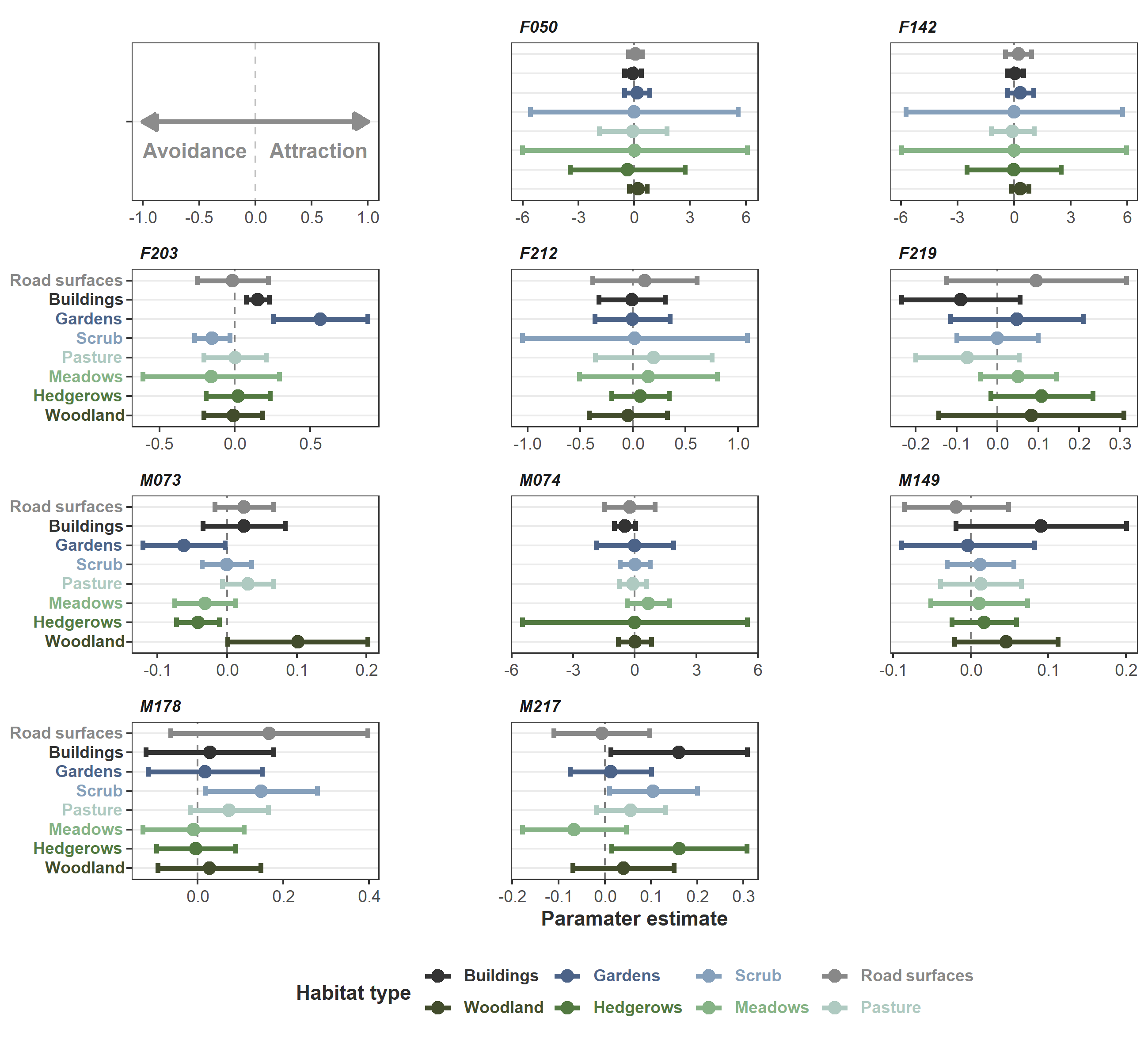

Supplement: S6 Fig — Each plot displays the habitat selection of one individual snake. Positive values indicate selection for a habitat type, while negative values indicate avoidance. Error bars are 95% confidence intervals. As these snakes were not range resident, we cannot have confidence in values for their parameter estimates. (TIF) [file pone.0310352.s006.tif]

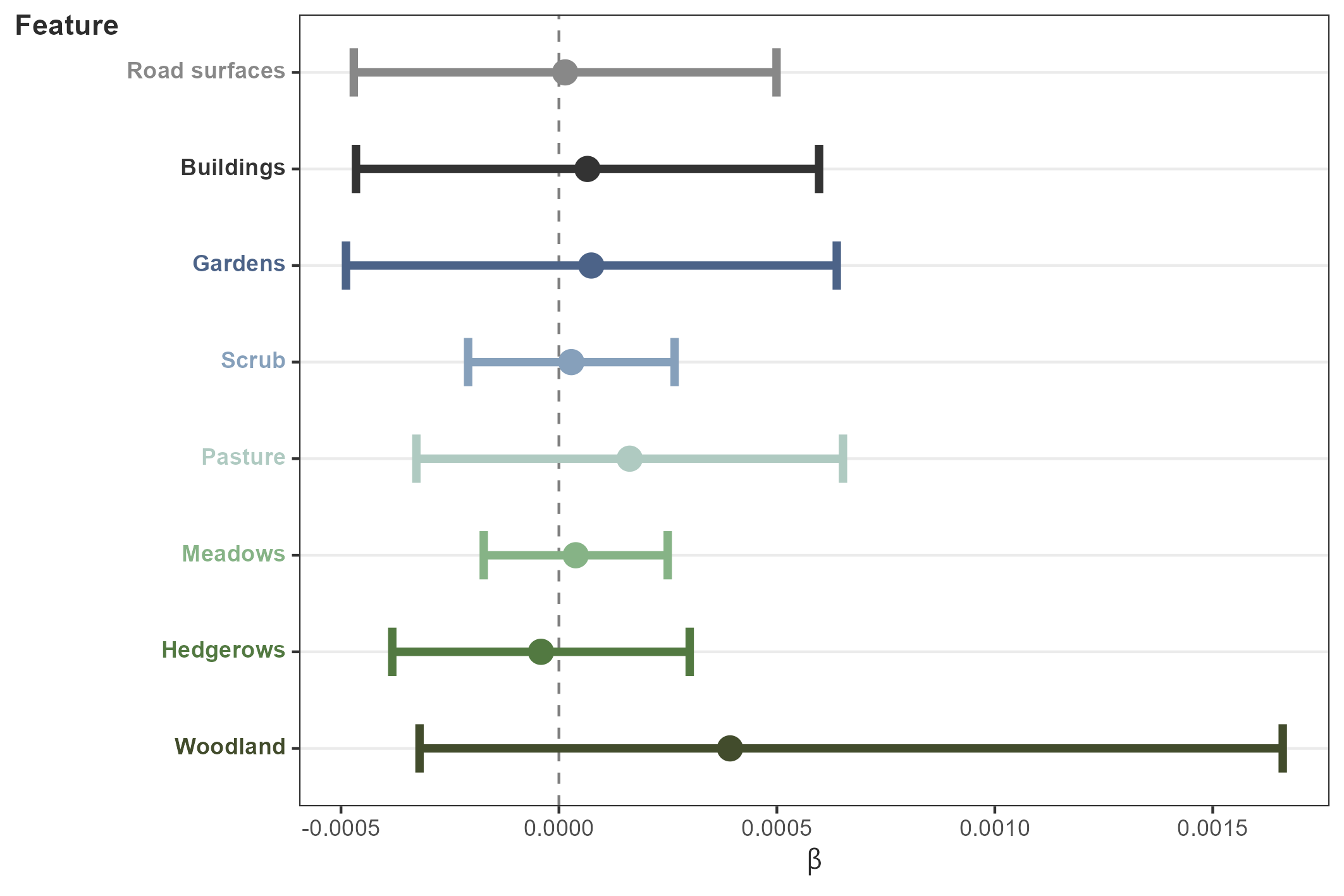

Supplement: S7 Fig — Error bars represent 99% confidence intervals. (TIF) [file pone.0310352.s007.tif]

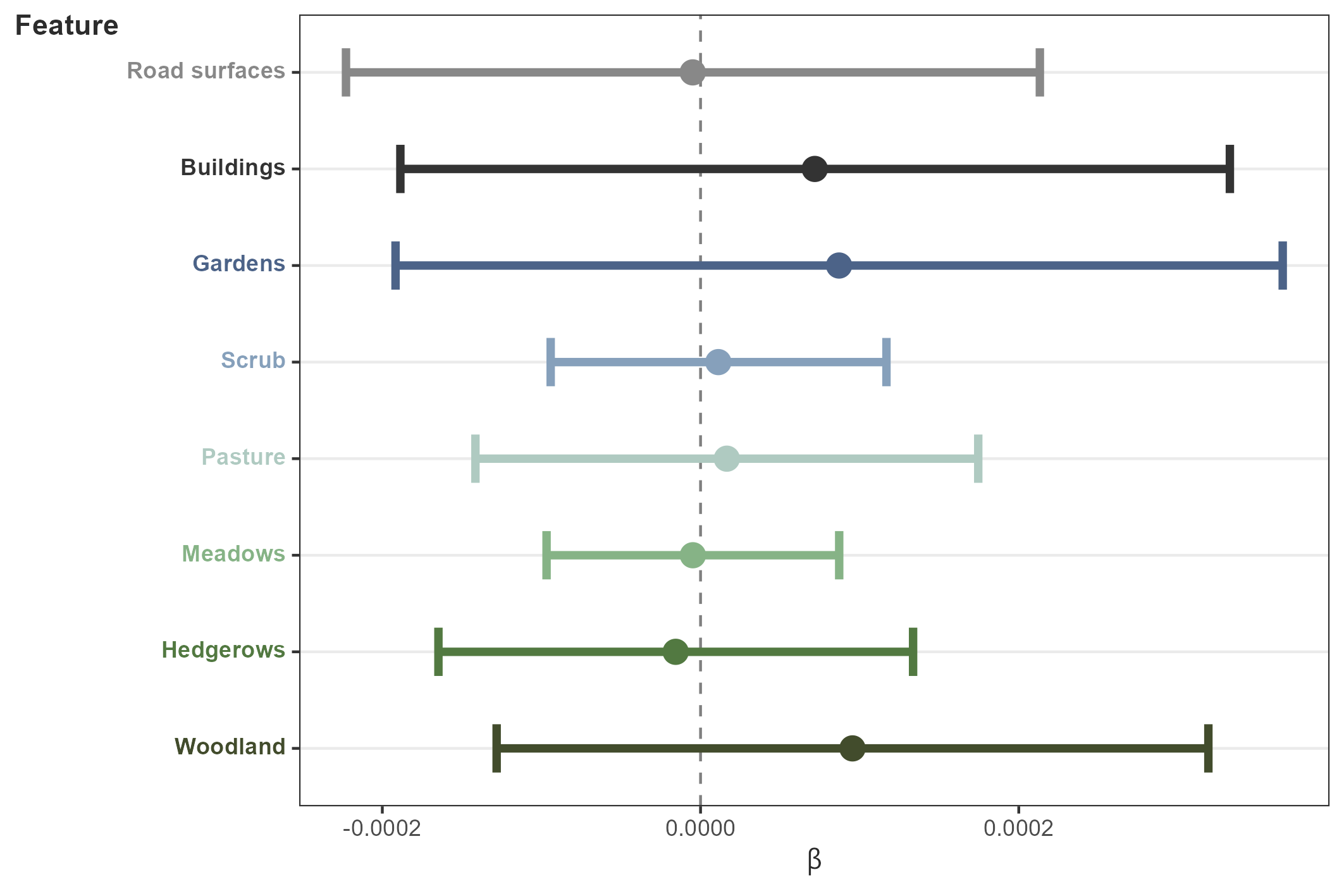

Supplement: S8 Fig — These results are from two daily tracks of all individuals. (TIF) [file pone.0310352.s008.tif]

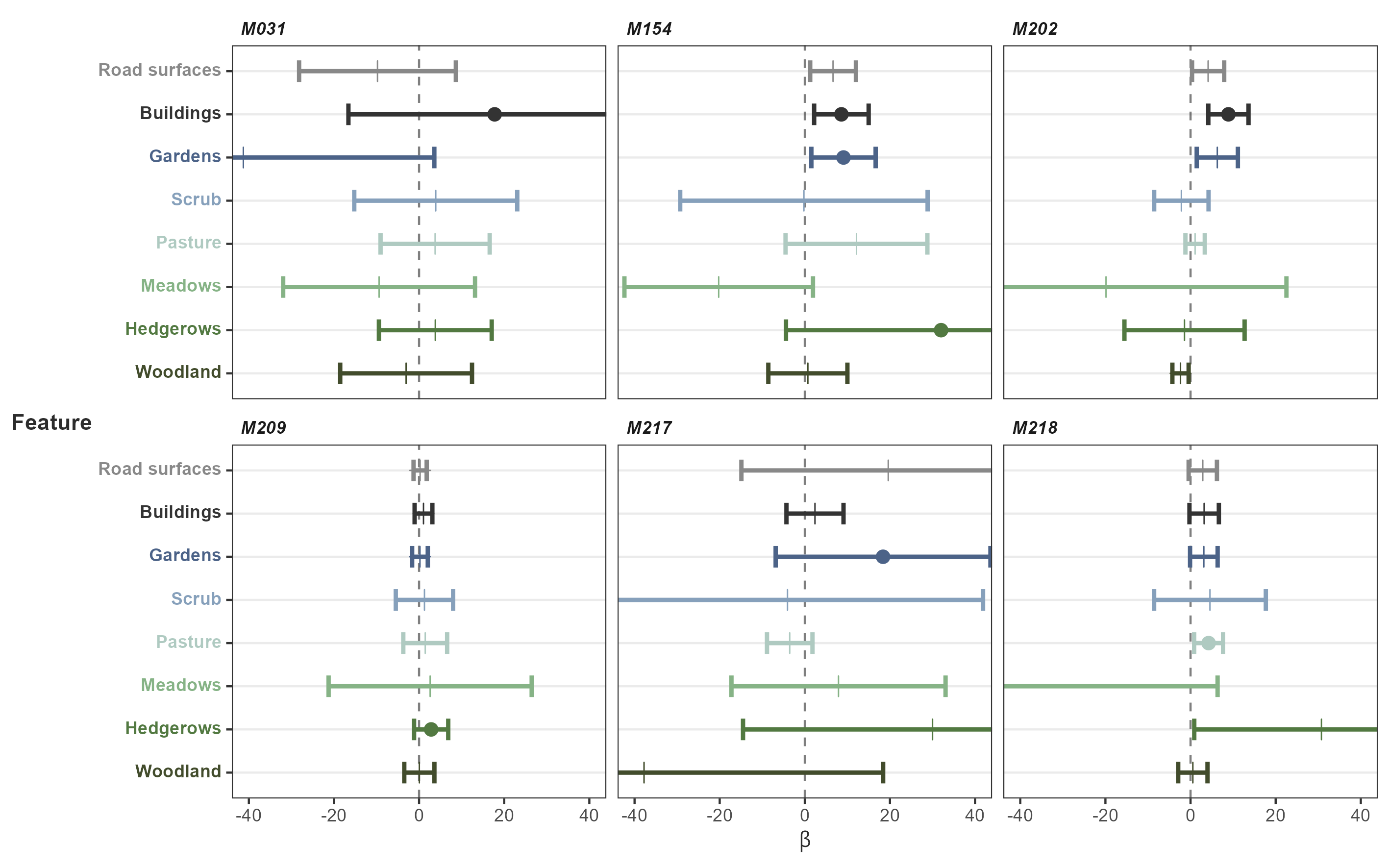

Supplement: S9 Fig — Positive values indicate a positive association with a habitat feature. Error bars indicate 95% confidence intervals. Circles indicate the features which were included in models with the lowest AIC score or scores < 2 greater. (TIF) [file pone.0310352.s009.tif]

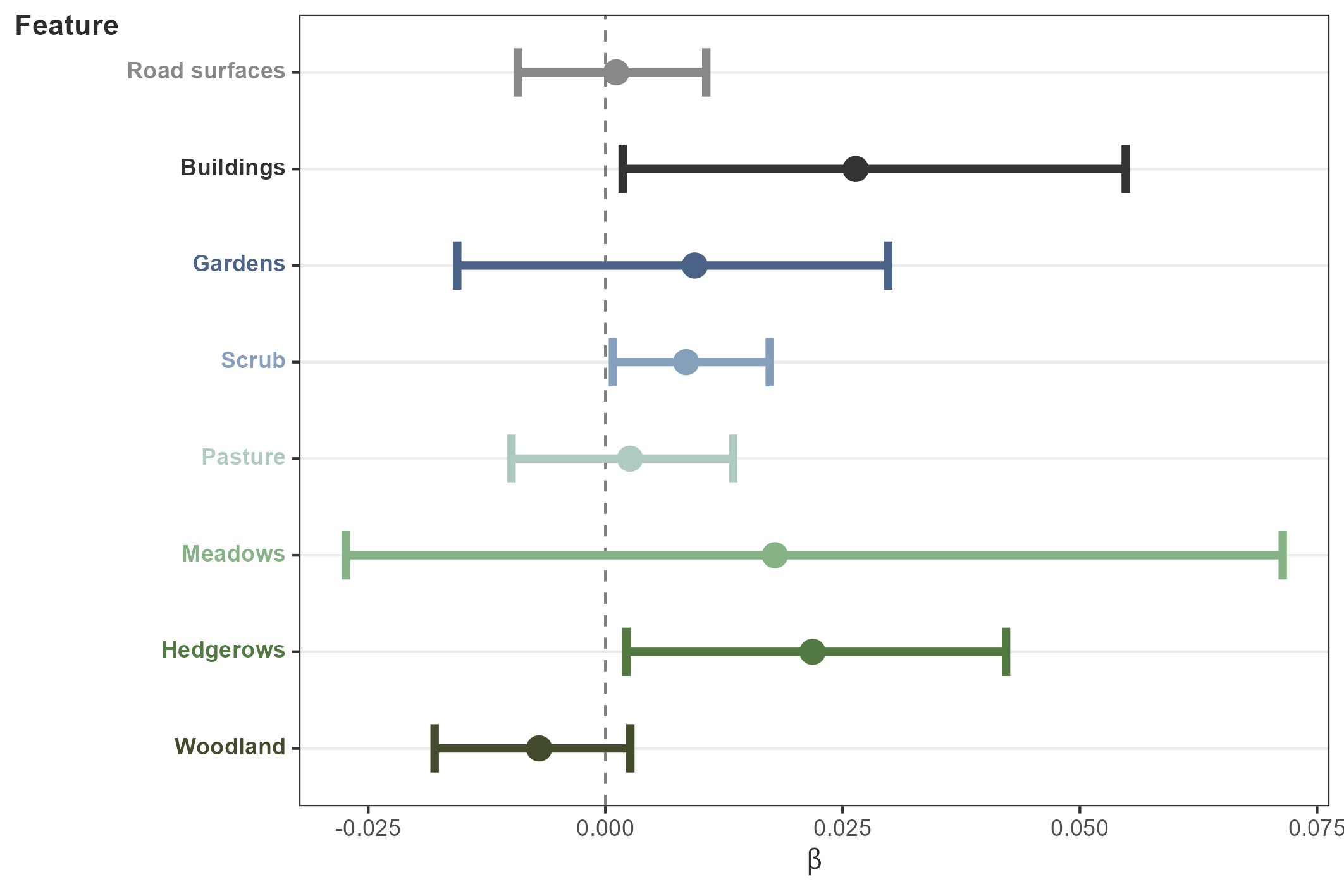

Supplement: S10 Fig — Positive values indicate a positive association with a habitat feature. Error bars indicate 95% confidence intervals. (TIF) [file pone.0310352.s010.tif]

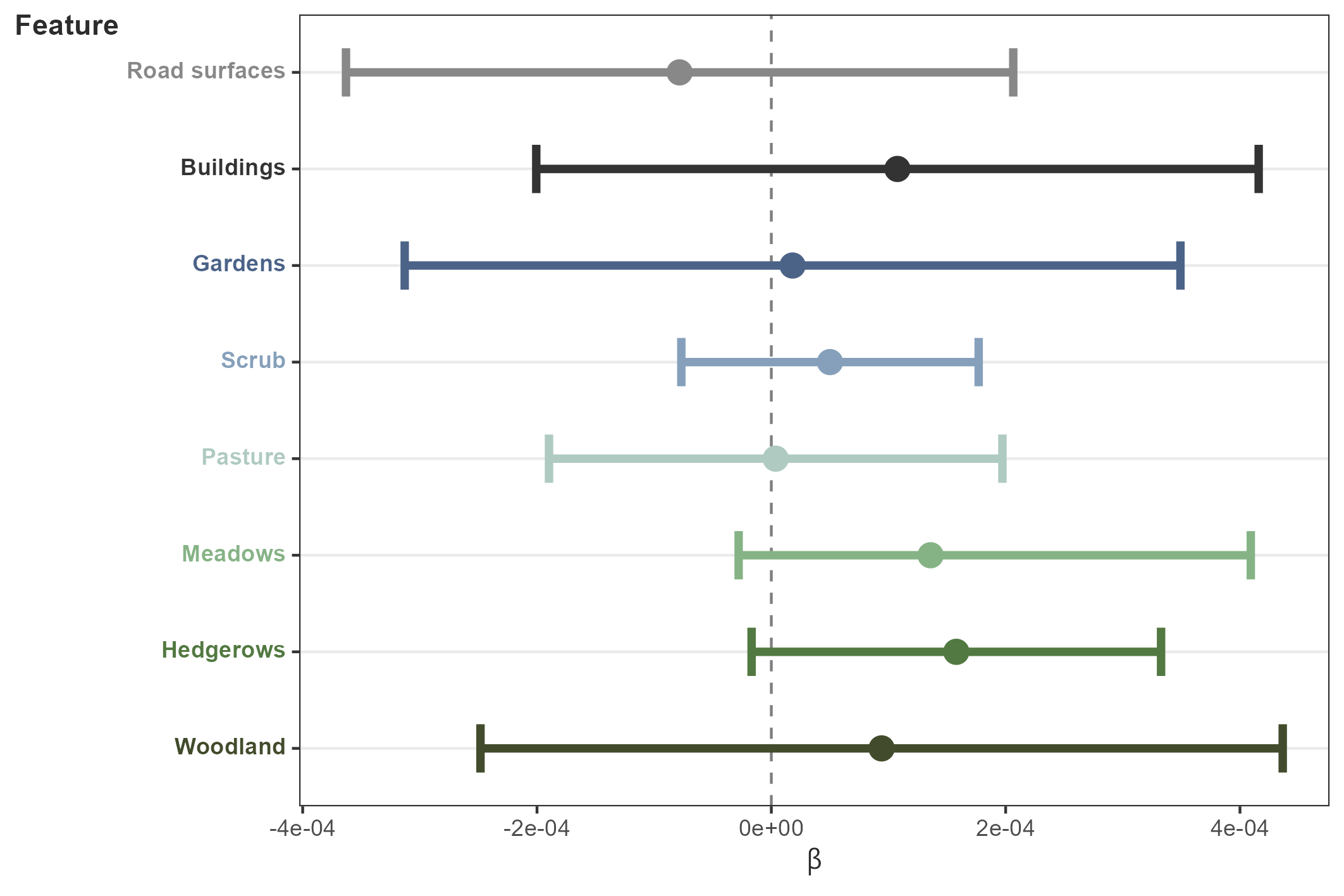

Supplement: S11 Fig — Error bars indicate 95% confidence intervals. (TIF) [file pone.0310352.s011.tif]

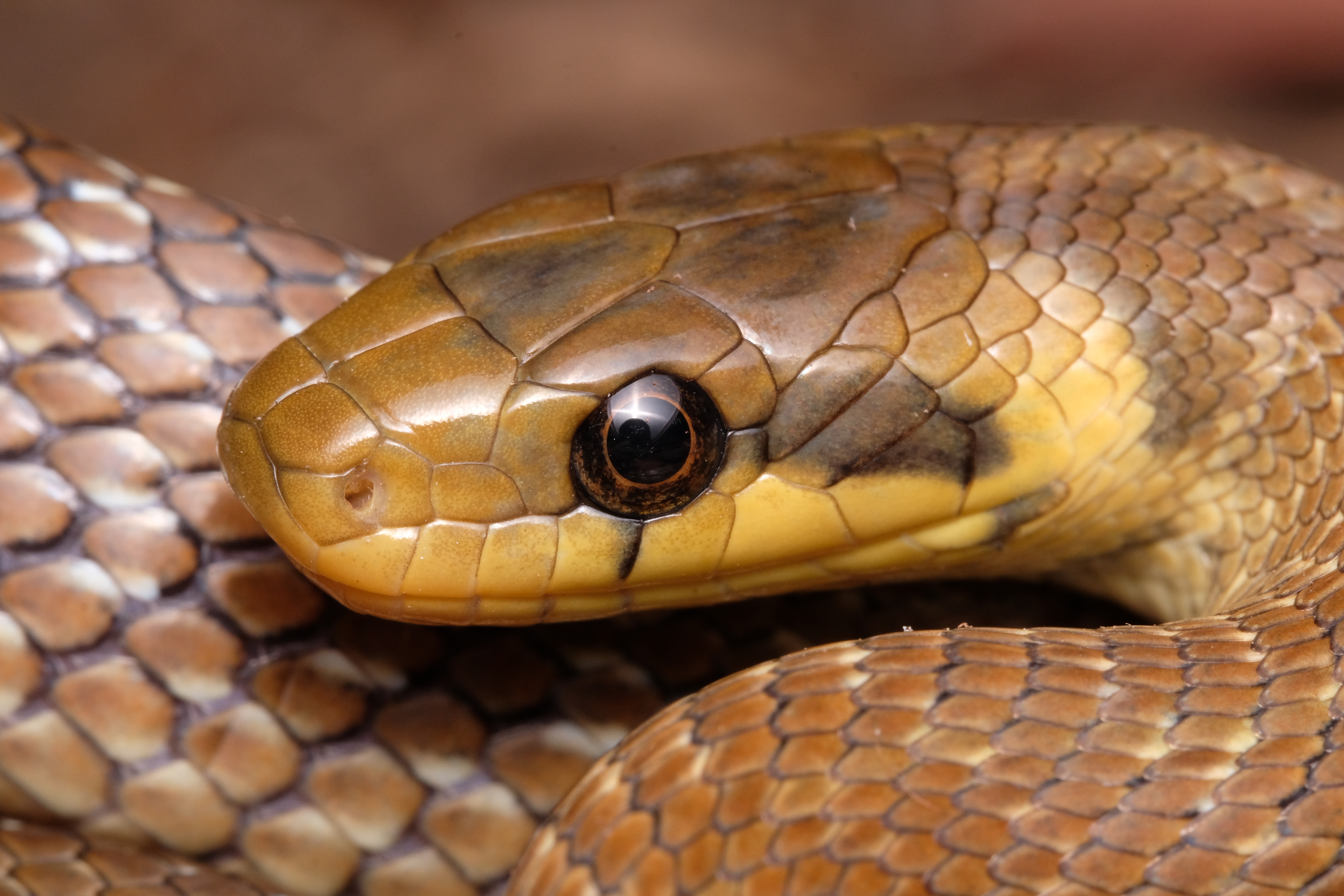

Supplement: S12 Fig — (JPG) [file pone.0310352.s012.JPG]

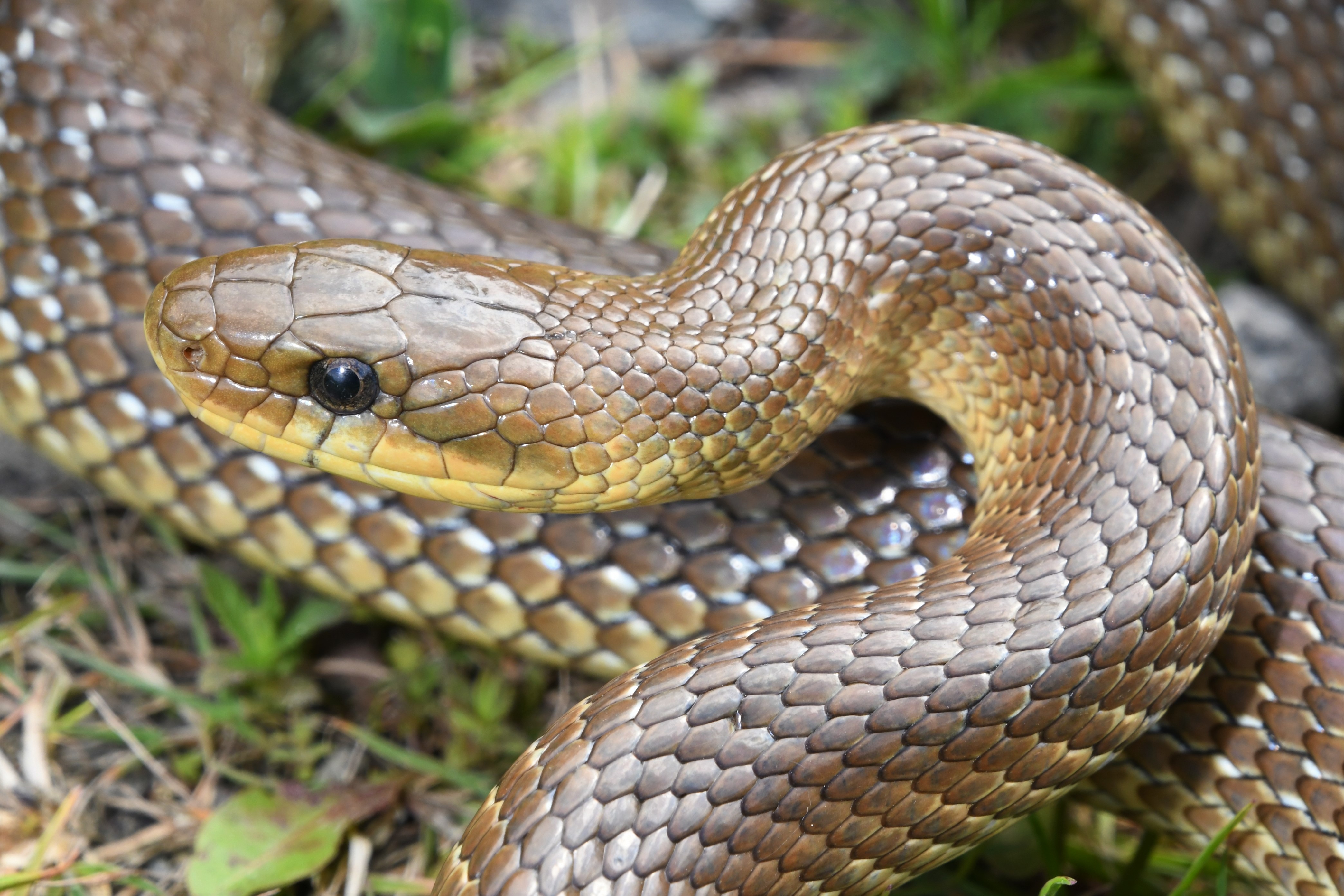

Supplement: S13 Fig — (JPG) [file pone.0310352.s013.JPG]

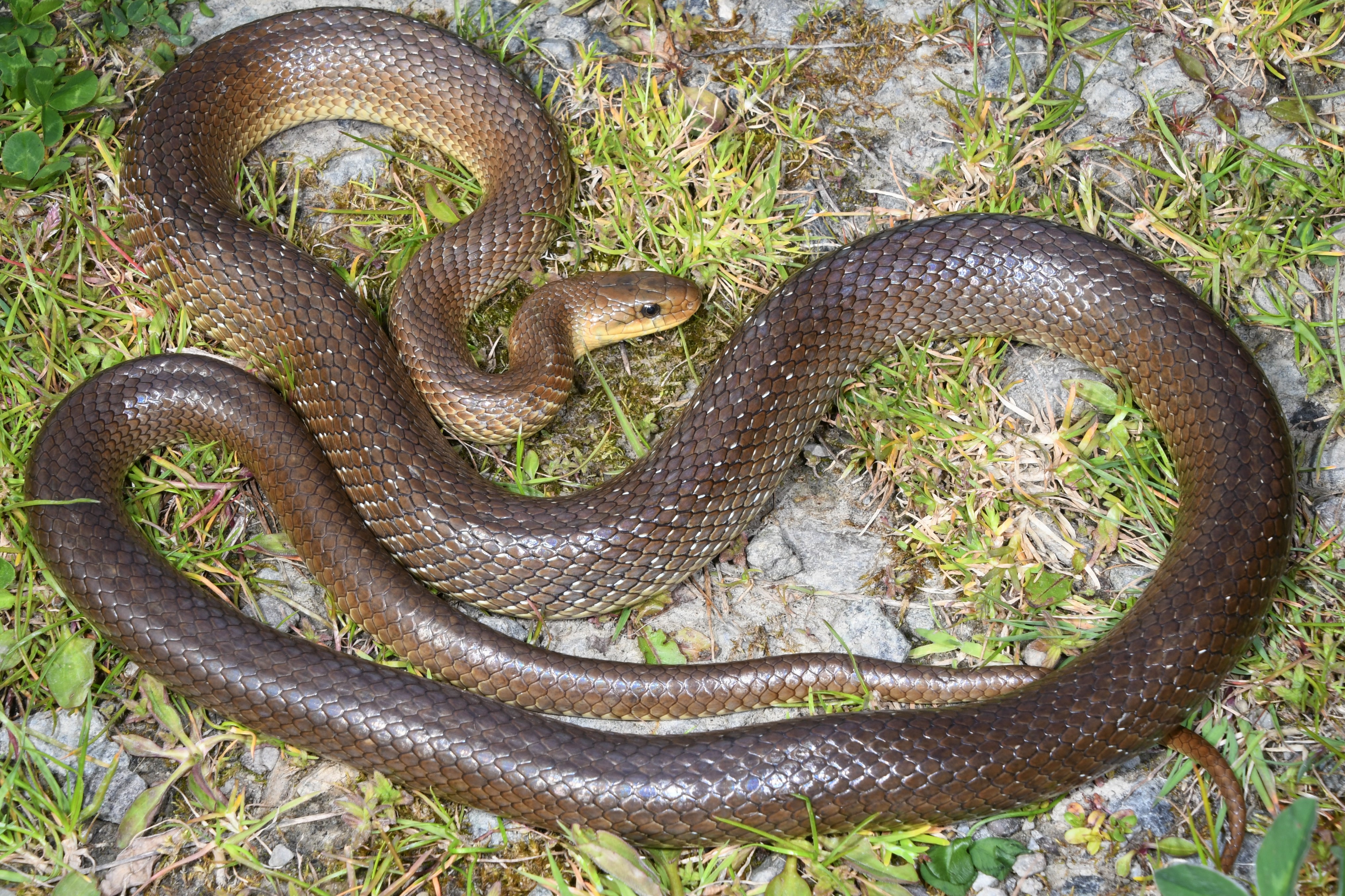

Supplement: S14 Fig — (JPG) [file pone.0310352.s014.JPG]
