# Supplementary material for: A reliance on human habitats is key to the success of an introduced predatory reptile
Source: PLoS One. 2025 Feb 5;20(2):e0310352. doi: 10.1371/journal.pone.0310352 (PMC11798526; doi:10.1371/journal.pone.0310352)
Supplement: S1 Table — MDD = mean daily displacement. (DOCX) [file pone.0310352.s015.docx]

| ID | Capture date | Released | Total distance moved (m) | MDD (m) | Maximum daily distance (m) | Relocations |
| --- | --- | --- | --- | --- | --- | --- |
| F050 | 13/06/2022 | 17/06/2022 | 216.20 | 7.74 | 56.78 | 13 |
| F142 | 24/05/2021 | 03/06/2021 | 264.26 | 8.05 | 58.09 | 20 |
| F158 | 22/06/2022 | 24/06/2022 | 1028.29 | 20.57 | 92.10 | 31 |
| F159 | 11/06/2021 | 18/06/2021 | 3673.04 | 60.38 | 364.43 | 47 |
| F177 | 07/07/2021 | 21/07/2021 | 782.51 | 8.68 | 44.67 | 47 |
| F203 | 11/05/2022 | 13/05/2022 | 1282.96 | 19.70 | 72.06 | 60 |
| F212 | 24/05/2022 | 27/05/2022 | 157.93 | 38.04 | 64.73 | 8 |
| F219 | 25/06/2022 | 29/06/2022 | 555.29 | 46.00 | 244.47 | 7 |
| M031 | 03/06/2022 | 07/06/2022 | 1135.56 | 34.20 | 129.11 | 45 |
| M073 | 10/05/2021 | 02/06/2021 | 698.28 | 78.71 | 206.39 | 8 |
| M074 | 14/07/2022 | 15/07/2022 | 179.99 | 5.97 | 64.96 | 6 |
| M137 | 10/05/2021 | 02/06/2021 | 3894.85 | 30.95 | 382.88 | 76 |
| M139 | 15/05/2021 | 03/06/2021 | 3903.90 | 41.10 | 559.30 | 64 |
| M149 | 02/06/2021 | 04/06/2021 | 1706.27 | 46.37 | 336.90 | 28 |
| M154 | 07/06/2022 | 17/06/2022 | 2427.55 | 32.70 | 107.16 | 75 |
| M178 | 25/07/2021 | 28/07/2021 | 999.82 | 12.04 | 255.31 | 25 |
| M180 | 29/07/2021 | 05/08/2021 | 1225.27 | 21.54 | 125.12 | 21 |
| M202 | 08/05/2022 | 11/05/2022 | 7935.06 | 70.85 | 566.25 | 142 |
| M209 | 20/05/2022 | 25/05/2022 | 7387.04 | 69.03 | 365.16 | 126 |
| M217 | 11/06/2022 | 14/06/2022 | 1477.92 | 86.94 | 383.95 | 23 |
| M218 | 21/06/2022 | 24/06/2022 | 4469.91 | 66.73 | 586.54 | 75 |
