# Supplementary material for: A reliance on human habitats is key to the success of an introduced predatory reptile
Source: PLoS One. 2025 Feb 5;20(2):e0310352. doi: 10.1371/journal.pone.0310352 (PMC11798526; doi:10.1371/journal.pone.0310352)
Supplement: S2 Table — Abbreviated capture types are mating with a tracked snake (mating), notification from the public (notification), and dedicated survey (survey). We did not record snout-vent length (SVL) for F203 in error. (DOCX) [file pone.0310352.s016.docx]

| ID | Mass (g) | SVL (mm) | Capture type | Start | End | Days | Fixes | Fate |
| --- | --- | --- | --- | --- | --- | --- | --- | --- |
| F050 | 208 | 766 | Opportunistic | 18/06/2022 | 16/07/2022 | 28 | 57 | Transmitter out of battery |
| F142 | 233 | 668 | Notification | 04/06/2021 | 07/07/2021 | 33 | 56 | Dead - car strike |
| F158 | 207 | 692 | Opportunistic | 25/06/2022 | 14/08/2022 | 50 | 101 | Transmitter out of battery |
| F159 | 266 | 845 | Opportunistic | 19/06/2021 | 19/08/2021 | 61 | 101 | Dead - eaten by M137 |
| F177 | 237 | 818 | Opportunistic | 22/07/2021 | 20/10/2021 | 90 | 128 | Transmitter out of battery in 2021. Dead - car strike in 2022 |
| F203 | 211 | - | Zookeeper | 14/05/2022 | 18/07/2022 | 65 | 216 | Transmitter out of battery |
| F212 | 343 | 785 | Mating | 31/05/2022 | 04/06/2022 | 4 | 23 | Transmitter malfunction |
| F219 | 322 | 872 | Opportunistic | 30/06/2022 | 12/07/2022 | 12 | 24 | Transmitter malfunction |
| M031 | 383 | 960 | Opportunistic | 08/06/2022 | 11/07/2022 | 33 | 164 | Dead - car strike |
| M073 | 395 | 1069 | Survey | 05/06/2021 | 14/06/2021 | 9 | 17 | Dead - buzzard predation |
| M074 | 399 | 1089 | Opportunistic | 16/07/2022 | 15/08/2022 | 30 | 144 | Transmitter out of battery |
| M137 | 512 | 1178 | Survey | 04/06/2021 | 08/10/2021 | 126 | 210 | Transmitter out of battery |
| M139 | 295 | 956 | Survey | 04/06/2021 | 07/09/2021 | 95 | 166 | Transmitter out of battery |
| M149 | 474 | 941 | Opportunistic | 05/06/2021 | 12/07/2021 | 37 | 62 | Dead - mammal predation |
| M154 | 398 | 971 | Opportunistic | 18/06/2022 | 31/08/2022 | 74 | 316 | Study period ended |
| M178 | 440 | 1052 | Opportunistic | 29/07/2021 | 20/10/2021 | 83 | 111 | Study period ended |
| M180 | 463 | 1011 | Notification | 06/08/2021 | 02/10/2021 | 57 | 95 | Study period ended |
| M202 | 498 | 1200 | Survey | 12/05/2022 | 31/08/2022 | 112 | 476 | Study period ended |
| M209 | 475 | 982 | Zookeeper | 16/05/2022 | 31/08/2022 | 107 | 438 | Study period ended |
| M217 | 235 | 801 | Opportunistic | 15/06/2022 | 01/07/2022 | 17 | 82 | Transmitter out of battery |
| M218 | 522 | 1115 | Opportunistic | 25/06/2022 | 31/08/2022 | 67 | 242 | Study period ended |
