# Supplementary material for: A reliance on human habitats is key to the success of an introduced predatory reptile
Source: PLoS One. 2025 Feb 5;20(2):e0310352. doi: 10.1371/journal.pone.0310352 (PMC11798526; doi:10.1371/journal.pone.0310352)
Supplement: S3 Table — Model abbreviations are as follows: OU = Ornstein-Uhlenbeck, OUF = Ornstein-Uhlenbeck foraging process, IID = independently and identically distributed. dRMSPE is the root mean squared prediction error. DOF area is the effective sample size. (DOCX) [file pone.0310352.s017.docx]

| ID | movMod | dAICc | dRMSPE (m) | DOF area |
| --- | --- | --- | --- | --- |
| F050 | OU anisotropic | 0.000 | 79.135 | 1.398 |
| F050 | OUF anisotropic | 1.929 | 44.597 | 3.139 |
| F050 | OUf anisotropic | 39.825 | 6.296 | 16.097 |
| F050 | OU | 57.092 | 11.335 | 4.040 |
| F050 | OUF | 59.052 | 6.893 | 8.309 |
| F050 | IID anisotropic | 121.699 | 0.000 | 56.000 |
| F142 | OU anisotropic | 0.000 | 30.813 | 1.857 |
| F142 | OUF anisotropic | 1.548 | 22.051 | 2.429 |
| F142 | OUf anisotropic | 5.364 | 3.333 | 16.888 |
| F142 | OU | 14.431 | 12.961 | 3.708 |
| F142 | OUF | 16.331 | 10.639 | 4.609 |
| F142 | IID anisotropic | 59.757 | 0.000 | 56.000 |
| F158 | OU | 0.000 | 11.896 | 8.719 |
| F158 | OUF | 1.936 | 6.396 | 14.030 |
| F158 | OU anisotropic | 2.750 | 11.884 | 8.806 |
| F158 | OUF anisotropic | 4.777 | 6.425 | 14.116 |
| F158 | OUf | 83.244 | 0.000 | 46.244 |
| F158 | OUf anisotropic | 83.727 | 0.704 | 45.317 |
| F158 | IID | 352.640 | 0.221 | 101.000 |
| F159 | OUF anisotropic | 0.000 | 62.952 | 5.986 |
| F159 | OUF | 3.888 | 92.126 | 5.695 |
| F159 | OUf anisotropic | 32.321 | 0.000 | 24.656 |
| F159 | OU anisotropic | 35.126 | 86.576 | 3.656 |
| F177 | OU anisotropic | 0.000 | 1.425 | 35.567 |
| F177 | OUF anisotropic | 2.106 | 1.049 | 41.296 |
| F177 | OU | 5.475 | 1.306 | 36.449 |
| F177 | OUF | 7.513 | 0.945 | 42.162 |
| F177 | OUf anisotropic | 55.202 | 1.125 | 72.027 |
| F177 | IID anisotropic | 226.211 | 0.000 | 125.000 |
| F203 | OU anisotropic | 0.000 | 0.000 | 27.028 |
| F203 | OUF anisotropic | 1.900 | 1.111 | 28.238 |
| F203 | OUf anisotropic | 6.727 | 20.652 | 39.683 |
| F203 | IID anisotropic | 59.275 | 65.023 | 65.000 |
| F203 | OU | 66.136 | 379.251 | 17.983 |
| F203 | OUF | 68.339 | 372.374 | 18.521 |
| F212 | OU anisotropic | 0.000 | 15.803 | 2.006 |
| F212 | OUf anisotropic | 0.391 | 0.000 | 7.678 |
| F212 | OUF anisotropic | 1.609 | 9.021 | 2.977 |
| F212 | IID anisotropic | 57.909 | 8.675 | 23.000 |
| F212 | OU | 72.455 | 51.705 | 1.439 |
| F212 | OUF | 73.870 | 41.980 | 1.738 |
| F219 | OU anisotropic | 0.000 | 65.082 | 2.363 |
| F219 | OUF anisotropic | 2.776 | 31.911 | 5.538 |
| F219 | OUf anisotropic | 5.548 | 15.829 | 7.386 |
| F219 | IID anisotropic | 21.476 | 0.000 | 25.000 |
| F219 | OU | 34.915 | 193.764 | 1.562 |
| F219 | OUF | 37.024 | 97.352 | 3.290 |
| M031 | OU anisotropic | 0.000 | 4.935 | 19.441 |
| M031 | OUF anisotropic | 2.009 | 2.932 | 25.168 |
| M031 | OU | 65.621 | 7.557 | 18.805 |
| M031 | OUF | 67.577 | 5.386 | 24.709 |
| M031 | OUf anisotropic | 80.786 | 0.089 | 44.489 |
| M031 | IID anisotropic | 485.599 | 0.000 | 164.000 |
| M073 | OUf anisotropic | 0.000 | 29.358 | 9.967 |
| M073 | OU anisotropic | 0.932 | 28.000 | 4.069 |
| M073 | OUF anisotropic | 5.670 | 0.000 | 0.000 |
| M073 | IID anisotropic | 22.338 | 24.694 | 17.000 |
| M073 | OUF | 57.510 | 45.703 | 5.752 |
| M073 | OUf | 65.971 | 29.656 | 10.357 |
| M074 | OUF anisotropic | 0.000 | 3.331 | 15.079 |
| M074 | OU anisotropic | 13.820 | 5.907 | 7.628 |
| M074 | OUf anisotropic | 19.072 | 0.000 | 44.979 |
| M074 | OUF | 166.274 | 2.059 | 58.139 |
| M137 | OU anisotropic | 0.000 | 38.972 | 9.360 |
| M137 | OUF anisotropic | 1.905 | 23.407 | 14.792 |
| M137 | OU | 197.305 | 107.496 | 6.067 |
| M137 | OUF | 199.126 | 79.049 | 10.967 |
| M137 | OUf anisotropic | 274.591 | 0.000 | 85.081 |
| M137 | IID anisotropic | 888.175 | 54.997 | 208.000 |
| M139 | OU anisotropic | 0.000 | 44.243 | 8.691 |
| M139 | OUF anisotropic | 1.924 | 26.855 | 13.932 |
| M139 | OUf anisotropic | 145.798 | 0.000 | 52.998 |
| M139 | OU | 251.646 | 139.605 | 5.271 |
| M139 | OUF | 253.592 | 138.154 | 5.455 |
| M139 | IID anisotropic | 550.045 | 78.689 | 166.000 |
| M149 | OU anisotropic | 0.000 | 16.780 | 8.905 |
| M149 | OUF anisotropic | 2.208 | 11.574 | 0.000 |
| M149 | OUf anisotropic | 17.924 | 0.000 | 33.490 |
| M149 | OU | 97.720 | 47.619 | 4.575 |
| M149 | OUF | 99.381 | 39.930 | 5.686 |
| M149 | IID anisotropic | 126.380 | 0.607 | 62.000 |
| M154 | OU anisotropic | 0.000 | 22.310 | 14.270 |
| M154 | OUF anisotropic | 1.913 | 16.506 | 20.202 |
| M154 | OU | 67.108 | 33.923 | 12.579 |
| M154 | OUF | 68.117 | 33.237 | 13.625 |
| M154 | OUf anisotropic | 354.253 | 0.000 | 76.143 |
| M154 | IID anisotropic | 1299.387 | 17.920 | 301.000 |
| M178 | OU anisotropic | 0.000 | 41.954 | 9.502 |
| M178 | OUF anisotropic | 2.000 | 35.395 | 15.055 |
| M178 | OUf anisotropic | 60.893 | 19.416 | 39.676 |
| M178 | OU | 132.452 | 10.928 | 18.604 |
| M178 | OUF | 134.438 | 8.579 | 25.770 |
| M178 | IID anisotropic | 165.861 | 0.000 | 108.000 |
| M180 | OU anisotropic | 0.000 | 3.652 | 14.887 |
| M180 | OUF anisotropic | 2.094 | 0.642 | 20.382 |
| M180 | OU | 45.310 | 17.582 | 10.909 |
| M180 | OUF | 47.282 | 12.577 | 16.344 |
| M180 | OUf anisotropic | 53.742 | 0.000 | 49.021 |
| M180 | IID anisotropic | 216.504 | 8.612 | 95.000 |
| M202 | OU anisotropic | 0.000 | 42.097 | 18.359 |
| M202 | OUF anisotropic | 1.911 | 31.826 | 24.517 |
| M202 | OU | 35.568 | 52.740 | 17.494 |
| M202 | OUF | 37.459 | 41.627 | 23.764 |
| M202 | OUf anisotropic | 851.468 | 0.000 | 127.852 |
| M202 | IID anisotropic | 1790.522 | 24.339 | 456.000 |
| M209 | OUF anisotropic | 0.000 | 38.160 | 20.562 |
| M209 | OU anisotropic | 55.661 | 41.911 | 14.858 |
| M209 | OUF | 67.018 | 56.547 | 19.221 |
| M209 | OUf anisotropic | 339.884 | 0.000 | 136.143 |
| M217 | OUF anisotropic | 0.000 | 103.607 | 1.837 |
| M217 | OU anisotropic | 2.957 | 128.102 | 1.582 |
| M217 | OUF | 36.950 | 190.779 | 1.589 |
| M217 | OUf anisotropic | 57.720 | 0.000 | 17.417 |
| M218 | OU anisotropic | 0.000 | 165.379 | 2.623 |
| M218 | OUF anisotropic | 2.066 | 164.682 | 2.652 |
| M218 | OU | 45.480 | 94.254 | 3.521 |
| M218 | OUF | 47.235 | 49.683 | 7.543 |
| M218 | OUf anisotropic | 251.001 | 37.463 | 47.286 |
| M218 | IID anisotropic | 1171.752 | 0.000 | 191.000 |
