# Supplementary material for: A reliance on human habitats is key to the success of an introduced predatory reptile
Source: PLoS One. 2025 Feb 5;20(2):e0310352. doi: 10.1371/journal.pone.0310352 (PMC11798526; doi:10.1371/journal.pone.0310352)
Supplement: S4 Table — Positive values denote selection for a particular habitat type, while negative values denote avoidance. (DOCX) [file pone.0310352.s018.docx]

| ID | Habitat | low | est | high |
| --- | --- | --- | --- | --- |
| F050 | Meadow | -6.02154 | 0.028014 | 6.077569 |
| F050 | Pasture | -1.89034 | -0.06947 | 1.751404 |
| F050 | Hedgerow | -3.45003 | -0.36169 | 2.726645 |
| F050 | Woodland | -0.2737 | 0.20705 | 0.687801 |
| F050 | Scrub | -5.57957 | -0.00101 | 5.577562 |
| F050 | Gardens | -0.51784 | 0.15483 | 0.827495 |
| F050 | Buildings | -0.53524 | -0.07972 | 0.375799 |
| F050 | Road surface | -0.30989 | 0.062338 | 0.434566 |
| F142 | Meadow | -5.96254 | 0.003173 | 5.968889 |
| F142 | Pasture | -1.21414 | -0.07342 | 1.0673 |
| F142 | Hedgerow | -2.49284 | -0.00378 | 2.485277 |
| F142 | Woodland | -0.13097 | 0.332156 | 0.795284 |
| F142 | Scrub | -5.72704 | 0.012234 | 5.751511 |
| F142 | Gardens | -0.3493 | 0.349517 | 1.048329 |
| F142 | Buildings | -0.35455 | 0.069545 | 0.493637 |
| F142 | Road surface | -0.45362 | 0.237717 | 0.929054 |
| F158 | Meadow | -0.13469 | -0.03771 | 0.059261 |
| F158 | Pasture | -0.03793 | 0.045544 | 0.129019 |
| F158 | Hedgerow | -0.03866 | 0.04395 | 0.126559 |
| F158 | Woodland | -0.12922 | -0.021 | 0.08722 |
| F158 | Scrub | -0.03193 | 0.070361 | 0.172648 |
| F158 | Gardens | -0.15358 | -0.00201 | 0.149556 |
| F158 | Buildings | -0.15582 | -0.00214 | 0.151545 |
| F158 | Road surface | -0.02279 | 0.139107 | 0.301007 |
| F159 | Meadow | -0.01885 | 0.012073 | 0.042993 |
| F159 | Pasture | -0.03312 | -0.01777 | -0.00243 |
| F159 | Hedgerow | -0.00828 | 0.022332 | 0.05294 |
| F159 | Woodland | -0.02117 | 0.011644 | 0.044458 |
| F159 | Scrub | -0.02252 | -0.00019 | 0.022149 |
| F159 | Gardens | -0.05646 | 0.000154 | 0.056766 |
| F159 | Buildings | -0.0311 | 0.035769 | 0.102636 |
| F159 | Road surface | -0.05721 | -0.01512 | 0.026964 |
| F177 | Meadow | -2.74367 | 0.000121 | 2.743911 |
| F177 | Pasture | -0.1614 | 0.090254 | 0.341904 |
| F177 | Hedgerow | -1.59735 | 0.184416 | 1.966181 |
| F177 | Woodland | -0.178 | -0.06913 | 0.039748 |
| F177 | Scrub | -3.29696 | 0.000421 | 3.297797 |
| F177 | Gardens | -0.14968 | 0.003336 | 0.156353 |
| F177 | Buildings | -0.21201 | -0.08476 | 0.042479 |
| F177 | Road surface | -0.13173 | 0.148447 | 0.42863 |
| F203 | Meadow | -0.60933 | -0.15694 | 0.295458 |
| F203 | Pasture | -0.20578 | 0.000431 | 0.206645 |
| F203 | Hedgerow | -0.19109 | 0.0209 | 0.232893 |
| F203 | Woodland | -0.20604 | -0.01115 | 0.183732 |
| F203 | Scrub | -0.26688 | -0.14997 | -0.03305 |
| F203 | Gardens | 0.253335 | 0.567464 | 0.881593 |
| F203 | Buildings | 0.074998 | 0.150827 | 0.226656 |
| F203 | Road surface | -0.24899 | -0.01443 | 0.220122 |
| F212 | Meadow | -0.50902 | 0.145556 | 0.80013 |
| F212 | Pasture | -0.35667 | 0.197284 | 0.75124 |
| F212 | Hedgerow | -0.20231 | 0.069918 | 0.342148 |
| F212 | Woodland | -0.41514 | -0.0445 | 0.326138 |
| F212 | Scrub | -1.05032 | 0.017905 | 1.08613 |
| F212 | Gardens | -0.36285 | -0.00429 | 0.354268 |
| F212 | Buildings | -0.32132 | -0.00833 | 0.304659 |
| F212 | Road surface | -0.38185 | 0.114268 | 0.610383 |
| F219 | Meadow | -0.04221 | 0.050833 | 0.143878 |
| F219 | Pasture | -0.20066 | -0.07412 | 0.052415 |
| F219 | Hedgerow | -0.01672 | 0.108406 | 0.233529 |
| F219 | Woodland | -0.14377 | 0.082967 | 0.309705 |
| F219 | Scrub | -0.09988 | -0.0001 | 0.099672 |
| F219 | Gardens | -0.11437 | 0.047482 | 0.209331 |
| F219 | Buildings | -0.23484 | -0.0901 | 0.054642 |
| F219 | Road surface | -0.1256 | 0.095271 | 0.31614 |
| M031 | Meadow | -0.04049 | 0.000782 | 0.042054 |
| M031 | Pasture | -0.2231 | -0.06984 | 0.083426 |
| M031 | Hedgerow | -0.01591 | 0.049039 | 0.113986 |
| M031 | Woodland | -0.11059 | -0.029 | 0.052603 |
| M031 | Scrub | -0.08144 | -5.48E-05 | 0.081331 |
| M031 | Gardens | -0.2343 | -0.02452 | 0.185263 |
| M031 | Buildings | 0.053132 | 0.183704 | 0.314276 |
| M031 | Road surface | -0.16979 | -0.11527 | -0.06074 |
| M073 | Meadow | -0.07538 | -0.0315 | 0.012379 |
| M073 | Pasture | -0.0067 | 0.029918 | 0.066539 |
| M073 | Hedgerow | -0.07269 | -0.04206 | -0.01142 |
| M073 | Woodland | 0.000518 | 0.10119 | 0.201862 |
| M073 | Scrub | -0.03609 | -0.00072 | 0.034645 |
| M073 | Gardens | -0.12077 | -0.06184 | -0.0029 |
| M073 | Buildings | -0.03491 | 0.024027 | 0.082965 |
| M073 | Road surface | -0.01749 | 0.024437 | 0.066359 |
| M074 | Meadow | -0.3751 | 0.654905 | 1.684909 |
| M074 | Pasture | -0.75834 | -0.09013 | 0.578084 |
| M074 | Hedgerow | -5.48128 | -0.00102 | 5.479245 |
| M074 | Woodland | -0.79758 | 0.005731 | 0.809047 |
| M074 | Scrub | -0.72682 | 0.005931 | 0.738681 |
| M074 | Gardens | -1.87285 | 0.003226 | 1.879302 |
| M074 | Buildings | -0.99025 | -0.47835 | 0.033555 |
| M074 | Road surface | -1.49104 | -0.25346 | 0.984112 |
| M137 | Meadow | -0.01307 | 0.004813 | 0.022699 |
| M137 | Pasture | -0.0083 | 0.004802 | 0.017901 |
| M137 | Hedgerow | -0.01533 | 0.005463 | 0.02626 |
| M137 | Woodland | 0.007771 | 0.060717 | 0.113663 |
| M137 | Scrub | -0.00914 | 0.004137 | 0.01741 |
| M137 | Gardens | -0.01937 | 0.046381 | 0.112134 |
| M137 | Buildings | -0.00013 | 0.054839 | 0.109806 |
| M137 | Road surface | -0.01442 | 0.017411 | 0.049239 |
| M139 | Meadow | -0.01207 | 0.006903 | 0.025878 |
| M139 | Pasture | -0.00628 | 0.006516 | 0.019312 |
| M139 | Hedgerow | -0.01635 | 0.005702 | 0.027751 |
| M139 | Woodland | 0.030819 | 0.123655 | 0.216491 |
| M139 | Scrub | -0.00502 | 0.007108 | 0.019234 |
| M139 | Gardens | -0.00057 | 0.098804 | 0.198182 |
| M139 | Buildings | 0.014586 | 0.092912 | 0.171238 |
| M139 | Road surface | -0.02239 | 0.0177 | 0.057788 |
| M149 | Meadow | -0.05162 | 0.010765 | 0.073156 |
| M149 | Pasture | -0.0392 | 0.012983 | 0.065163 |
| M149 | Hedgerow | -0.02454 | 0.016969 | 0.058478 |
| M149 | Woodland | -0.02092 | 0.045743 | 0.112407 |
| M149 | Scrub | -0.0308 | 0.01216 | 0.05512 |
| M149 | Gardens | -0.08922 | -0.00368 | 0.081867 |
| M149 | Buildings | -0.0194 | 0.090623 | 0.200649 |
| M149 | Road surface | -0.08577 | -0.01856 | 0.048647 |
| M154 | Meadow | -0.2226 | -0.06822 | 0.086161 |
| M154 | Pasture | -0.04825 | 0.012208 | 0.072665 |
| M154 | Hedgerow | 0.026308 | 0.124188 | 0.222069 |
| M154 | Woodland | -0.13756 | -0.05274 | 0.032084 |
| M154 | Scrub | -0.07906 | -0.00442 | 0.070213 |
| M154 | Gardens | -0.10168 | 0.001377 | 0.104437 |
| M154 | Buildings | -0.00296 | 0.0823 | 0.167558 |
| M154 | Road surface | -0.10783 | 0.003547 | 0.114922 |
| M178 | Meadow | -0.12805 | -0.01007 | 0.107903 |
| M178 | Pasture | -0.01848 | 0.072852 | 0.164183 |
| M178 | Hedgerow | -0.09658 | -0.00424 | 0.088102 |
| M178 | Woodland | -0.09325 | 0.027055 | 0.14736 |
| M178 | Scrub | 0.017274 | 0.148056 | 0.278838 |
| M178 | Gardens | -0.11621 | 0.01689 | 0.149993 |
| M178 | Buildings | -0.12157 | 0.027974 | 0.177516 |
| M178 | Road surface | -0.06291 | 0.167217 | 0.39734 |
| M180 | Meadow | -0.06384 | 0.021628 | 0.107092 |
| M180 | Pasture | -0.14144 | -0.03803 | 0.065381 |
| M180 | Hedgerow | -0.07601 | -0.00379 | 0.068441 |
| M180 | Woodland | -0.11047 | -0.05155 | 0.007382 |
| M180 | Scrub | -0.07513 | -0.00133 | 0.072468 |
| M180 | Gardens | 0.078996 | 0.219049 | 0.359103 |
| M180 | Buildings | 0.154185 | 0.351036 | 0.547887 |
| M180 | Road surface | -0.65745 | -0.43359 | -0.20973 |
| M202 | Meadow | 0.048305 | 0.074868 | 0.10143 |
| M202 | Pasture | 0.004216 | 0.01226 | 0.020303 |
| M202 | Hedgerow | -0.00467 | 0.014752 | 0.034175 |
| M202 | Woodland | -0.04131 | -0.02568 | -0.01006 |
| M202 | Scrub | -0.01701 | -0.00304 | 0.010928 |
| M202 | Gardens | -0.07003 | -0.03322 | 0.003593 |
| M202 | Buildings | 0.107229 | 0.147704 | 0.188178 |
| M202 | Road surface | -0.06149 | -0.03989 | -0.01828 |
| M209 | Meadow | -0.00284 | 0.013807 | 0.030458 |
| M209 | Pasture | 0.006265 | 0.018235 | 0.030204 |
| M209 | Hedgerow | -0.00605 | 0.014013 | 0.034073 |
| M209 | Woodland | -0.02419 | -0.00727 | 0.009646 |
| M209 | Scrub | -0.03137 | -0.0154 | 0.000572 |
| M209 | Gardens | -0.04728 | -0.01747 | 0.012351 |
| M209 | Buildings | 0.008896 | 0.038325 | 0.067753 |
| M209 | Road surface | -0.00198 | 0.01614 | 0.034263 |
| M217 | Meadow | -0.17865 | -0.06621 | 0.046231 |
| M217 | Pasture | -0.01869 | 0.056179 | 0.131046 |
| M217 | Hedgerow | 0.014308 | 0.160708 | 0.307107 |
| M217 | Woodland | -0.06897 | 0.040133 | 0.149235 |
| M217 | Scrub | 0.009633 | 0.104708 | 0.199782 |
| M217 | Gardens | -0.07508 | 0.012765 | 0.10061 |
| M217 | Buildings | 0.012771 | 0.160424 | 0.308077 |
| M217 | Road surface | -0.11006 | -0.00676 | 0.09654 |
| M218 | Meadow | -0.01615 | 0.041303 | 0.098755 |
| M218 | Pasture | -0.01699 | 0.008912 | 0.034818 |
| M218 | Hedgerow | -0.01138 | 0.042477 | 0.096332 |
| M218 | Woodland | -0.0146 | 0.017738 | 0.050079 |
| M218 | Scrub | -0.03115 | -0.0018 | 0.027543 |
| M218 | Gardens | -0.0974 | -0.02708 | 0.043252 |
| M218 | Buildings | -0.02948 | 0.049926 | 0.129328 |
| M218 | Road surface | -0.03538 | 0.015449 | 0.066282 |
