# Supplementary material for: A reliance on human habitats is key to the success of an introduced predatory reptile
Source: PLoS One. 2025 Feb 5;20(2):e0310352. doi: 10.1371/journal.pone.0310352 (PMC11798526; doi:10.1371/journal.pone.0310352)
Supplement: S5 Table — Asterisks mark the top performing model based on AIC scores and models with an AIC score < 2 from the top model. All models included the association of habitat with step length and turn angle. (DOCX) [file pone.0310352.s019.docx]

| Model | M031 | M154 | M202 | M209 | M217 | M218 |
| --- | --- | --- | --- | --- | --- | --- |
| Model 1 (null) | 259.46 | 511.17 | 1043.41 | 824.54 | 163.29 | 281.24 |
| Model 2 (Meadow) | 264.4 | 513.94 | 1027.49 | 821.71 | 168.47 | 283.73 |
| Model 3 (Pasture) | 264.96 | 510.94 | 1044.37 | 825.82 | 167.03 | *276.07* |
| Model 4 (Hedgerow) | 265.32 | *501.26* | 1039.91 | *816.42* | 163.13 | 279.42 |
| Model 5 (Woodland) | 264.74 | 514.83 | 1037 | 830 | 165.52 | 285.85 |
| Model 6 (Scrub) | 265.24 | 505.65 | 1042.47 | 828.2 | 166.24 | 284.26 |
| Model 7 (Gardens) | 256.83 | *502.87* | 1033.16 | 829.98 | *160.62* | 281.94 |
| Model 8 (Buildings) | *251.15* | *502.16* | *1018.96* | 829.41 | 165.65 | 282.59 |
| Model 9 (Road Surface) | 262.54 | 510.7 | 1041.14 | 830.44 | 164.02 | 283.12 |
