# Supplementary material for: A reliance on human habitats is key to the success of an introduced predatory reptile
Source: PLoS One. 2025 Feb 5;20(2):e0310352. doi: 10.1371/journal.pone.0310352 (PMC11798526; doi:10.1371/journal.pone.0310352)
Supplement: S6 Table — (DOCX) [file pone.0310352.s020.docx]

| ID | Mean time spent stationary (days) | Minimum time spent stationary (days) | Max time spent stationary (days) |
| --- | --- | --- | --- |
| F050 | 14.27 ± 1.17 | 0.11 | 20.14 |
| F142 | 3.49 ± 0.32 | 0.03 | 19.47 |
| F158 | 3.37 ± 0.23 | 0.06 | 11.06 |
| F159 | 3.37 ± 0.3 | 0.02 | 11.44 |
| F177 | 3.18 ± 0.22 | 0.1 | 18.9 |
| F203 | 3.01 ± 0.12 | 0.04 | 11.28 |
| F212 | 0.5 ± 0.06 | 0.01 | 1.91 |
| F219 | 8.1 ± 0.78 | 0.005 | 10 |
| M031 | 3.78 ± 0.53 | 0.04 | 14.6 |
| M073 | 1.3 ± 0.22 | 0.03 | 2.84 |
| M074 | 27.61 ± 0.81 | 0.09 | 29.02 |
| M137 | 5.26 ± 0.23 | 0.02 | 15.51 |
| M139 | 4.31 ± 0.37 | 0.02 | 17.01 |
| M149 | 2.83 ± 0.36 | 0.04 | 8.52 |
| M154 | 2.34 ± 0.17 | 0.02 | 13.73 |
| M178 | 5.17 ± 0.32 | 0.004 | 18.56 |
| M180 | 5.94 ± 0.52 | 0.01 | 16.1 |
| M202 | 1.98 ± 0.08 | 0.02 | 9.26 |
| M209 | 3.77 ± 0.24 | 0.02 | 13 |
| M217 | 0.82 ± 0.06 | 0.02 | 5.1 |
| M218 | 1.67 ± 0.09 | 0.02 | 7.39 |
