# Supplementary material for: A reliance on human habitats is key to the success of an introduced predatory reptile
Source: PLoS One. 2025 Feb 5;20(2):e0310352. doi: 10.1371/journal.pone.0310352 (PMC11798526; doi:10.1371/journal.pone.0310352)
Supplement: Attachment — Submitted filename: PONE-D-24-37502_WRS_20240919.pdf [file pone.0310352.s021.pdf]

PLOS ONE

A reliance on human habitats is key to the success of an introduced predatory reptile

--Manuscript Draft--

**Manuscript Number:**	PONE-D-24-37502
**Article Type:**	Research Article
**Full Title:**	A reliance on human habitats is key to the success of an introduced predatory reptile
**Short Title:**	Aesculapian snakes use human habitats in their introduced range
**Corresponding Author:**	Tom Major<br>Bournemouth University<br>Poole, Dorset UNITED KINGDOM OF GREAT BRITAIN AND NORTHERN IRELAND
**Keywords:**	Aesculapian, snake, Colubridae, spatial ecology
**Abstract:**	Understanding the success of animals in novel environments is increasingly important as human-mediated introductions continue to move species far beyond their natural ranges. Alongside these introductions, inhabited and agricultural areas are spreading, and correspondingly most animal introductions occur in populated areas. Commensal species which can live alongside humans by making use of specific conditions, structures, or prey, have a significant advantage. Introduced mammal species often use anthropogenic features in their environment and demonstrate a higher tolerance of human disturbance, but their importance remains understudied in ectotherms. The Aesculapian snake (<i>Zamenis longissimus</i>) is an ectotherm which has been introduced beyond the northern extremities of its natural range. To understand their persistence, we radio-tracked snakes daily over two active seasons, including high-frequency tracking of a subset of males. We investigated snake home range size using Autocorrelated Kernel Density Estimators (AKDE). Using AKDE-weighted Habitat Selection Functions we identified preferences for habitat features in a mosaic of habitats, and we used Integrated Step Selection Functions to further explore how these features influence movement. We revealed a particular preference for buildings in male snakes, while females preferred woodland. We demonstrate that the success of this ectothermic predator is likely tied to a willingness to use human features of the landscape.
**Order of Authors:**	Tom Major
	Lauren Jeffrey
	Guillem Limia Russel
	Rebecca Bracegirdle
	Antonio Gandini
	Rhys Morgan
	Benjamin Michael Marshall
	John F Mulley
	Wolfgang Wüster
**Additional Information:**	
**Question**	**Response**
**Financial Disclosure**<br><br>Enter a financial disclosure statement that describes the sources of funding for the work included in this submission. Review the submission guidelines for detailed requirements. View published research articles from *PLOS ONE* for specific	Yes

Powered by Editorial Manager® and ProduXion Manager® from Aries Systems Corporation

examples.<br><br>This statement is required for submission and **will appear in the published article** if the submission is accepted. Please make sure it is accurate.<br><br>**Funded studies**<br>Enter a statement with the following details:<br>• Initials of the authors who received each award<br>• Grant numbers awarded to each author<br>• The full name of each funder<br>• URL of each funder website<br>• Did the sponsors or funders play any role in the study design, data collection and analysis, decision to publish, or preparation of the manuscript?<br><br>Did you receive funding for this work?	
Please add funding details.<br>as follow-up to "**Financial Disclosure**<br><br>Enter a financial disclosure statement that describes the sources of funding for the work included in this submission. Review the submission guidelines for detailed requirements. View published research articles from *PLOS ONE* for specific examples.<br><br>This statement is required for submission and **will appear in the published article** if the submission is accepted. Please make sure it is accurate.<br><br>**Funded studies**<br>Enter a statement with the following details:<br>• Initials of the authors who received each award<br>• Grant numbers awarded to each author<br>• The full name of each funder<br>• URL of each funder website<br>• Did the sponsors or funders play any role in the study design, data collection and analysis, decision to publish, or preparation of the manuscript?	This work was supported by the Knowledge Economy Skills Scholarships (KESS II – case number 80815) and the Welsh Mountain Zoo, awarded to WW and JM for TM's PhD. Additional funding was provided by the Biodiversity and Ecosystem Evidence and Research Needs (BEERN) Programme awarded to TM.

Did you receive funding for this work?"	
Please select the country of your main research funder (please select carefully as in some cases this is used in fee calculation).<br>as follow-up to "**Financial Disclosure**<br><br>Enter a financial disclosure statement that describes the sources of funding for the work included in this submission. Review the submission guidelines for detailed requirements. View published research articles from *PLOS ONE* for specific examples.<br><br>This statement is required for submission and **will appear in the published article** if the submission is accepted. Please make sure it is accurate.<br><br>**Funded studies**<br>Enter a statement with the following details:<br>• Initials of the authors who received each award<br>• Grant numbers awarded to each author<br>• The full name of each funder<br>• URL of each funder website<br>• Did the sponsors or funders play any role in the study design, data collection and analysis, decision to publish, or preparation of the manuscript?<br><br>Did you receive funding for this work?"	UNITED KINGDOM - GB
**Competing Interests**<br><br>Use the instructions below to enter a competing interest statement for this submission. On behalf of all authors, disclose any competing interests that could be perceived to bias this work—acknowledging all financial support and any other relevant financial or non-financial competing interests.	The authors have declared that no competing interests exist.

This statement is required for submission and **will appear in the published article** if the submission is accepted. Please make sure it is accurate and that any funding sources listed in your Funding Information later in the submission form are also declared in your Financial Disclosure statement.<br><br>View published research articles from *PLOS ONE* for specific examples.<br><br>**NO authors have competing interests**<br><br>Enter: *The authors have declared that no competing interests exist.*<br><br>**Authors with competing interests**<br><br>Enter competing interest details beginning with this statement:<br><br>*I have read the journal's policy and the authors of this manuscript have the following competing interests: [insert competing interests here]*<br><br>* typeset	
**Ethics Statement**<br><br>Enter an ethics statement for this submission. This statement is required if the study involved:<br><br>• Human participants<br>• Human specimens or tissue<br>• Vertebrate animals or cephalopods<br>• Vertebrate embryos or tissues<br>• Field research<br><br>Write "N/A" if the submission does not require an ethics statement.<br><br>General guidance is provided below. Consult the submission guidelines for detailed instructions. **Make sure that all information entered here is included in the Methods section of the manuscript.**	This work took place with approval from the Bangor University Animal Welfare and Ethics Review Board. Telemetry procedures were conducted under Home Office project licence PC44793B6.

Powered by Editorial Manager® and ProduXion Manager® from Aries Systems Corporation

**Format for specific study types**<br><br>**Human Subject Research (involving human participants and/or tissue)**<br>• Give the name of the institutional review board or ethics committee that approved the study<br>• Include the approval number and/or a statement indicating approval of this research<br>• Indicate the form of consent obtained (written/oral) or the reason that consent was not obtained (e.g. the data were analyzed anonymously)<br><br>**Animal Research (involving vertebrate animals, embryos or tissues)**<br>• Provide the name of the Institutional Animal Care and Use Committee (IACUC) or other relevant ethics board that reviewed the study protocol, and indicate whether they approved this research or granted a formal waiver of ethical approval<br>• Include an approval number if one was obtained<br>• If the study involved *non-human primates*, add *additional details* about animal welfare and steps taken to ameliorate suffering<br>• If anesthesia, euthanasia, or any kind of animal sacrifice is part of the study, include briefly which substances and/or methods were applied<br><br>**Field Research**<br><br>Include the following details if this study involves the collection of plant, animal, or other materials from a natural setting:<br>• Field permit number<br>• Name of the institution or relevant body that granted permission	
**Data Availability**<br><br>Authors are required to make all data underlying the findings described fully available, without restriction, and from the time of publication. PLOS allows rare exceptions to address legal and ethical concerns. See the PLOS Data Policy and FAQ for detailed information.	Yes - all data are fully available without restriction

A Data Availability Statement describing where the data can be found is required at submission. Your answers to this question constitute the Data Availability Statement and **will be published in the article**, if accepted.<br><br>**Important:** Stating 'data available on request from the author' is not sufficient. If your data are only available upon request, select 'No' for the first question and explain your exceptional situation in the text box.<br><br>Do the authors confirm that all data underlying the findings described in their manuscript are fully available without restriction?	
**Describe where the data may be found in full sentences. If you are copying our sample text, replace any instances of XXX with the appropriate details.**<br><br>• If the data are **held or will be held in a public repository**, include URLs, accession numbers or DOIs. If this information will only be available after acceptance, indicate this by ticking the box below. For example: *All XXX files are available from the XXX database (accession number(s) XXX, XXX.).*<br>• If the data are all contained **within the manuscript and/or Supporting Information files**, enter the following: *All relevant data are within the manuscript and its Supporting Information files.*<br>• If neither of these applies but you are able to provide **details of access elsewhere**, with or without limitations, please do so. For example:<br><br>*Data cannot be shared publicly because of [XXX]. Data are available from the XXX Institutional Data Access / Ethics Committee (contact via XXX) for researchers who meet the criteria for access to confidential data.*<br><br>*The data underlying the results presented in the study are available*	All data and code are available from https://figshare.com/s/10b8bafb3ce0069555a1

from (include the name of the third party and contact information or URL).<br>• This text is appropriate if the data are owned by a third party and authors do not have permission to share the data.<br><br>* typeset	
Additional data availability information:	Tick here if the URLs/accession numbers/DOIs will be available only after acceptance of the manuscript for publication so that we can ensure their inclusion before publication.

Powered by Editorial Manager® and ProduXion Manager® from Aries Systems Corporation

Manuscript Click here to access/download;Manuscript;MS_Submission.docx

A reliance on human habitats is key to the success of an introduced predatory reptile

Tom Major[1], Lauren Jeffrey[2], Guillem Limia Russel[2], Rebecca Bracegirdle[2], Antonio Gandini[2], Rhys Morgan[2], Benjamin Michael Marshall[3], John F. Mulley[2], and Wolfgang Wüster[2]

[1] Department of Life & Environmental Sciences, Bournemouth University, Fern Barrow, Poole, Dorset, BH12 5BB, UK

[2] School of Environmental and Natural Sciences, Bangor University, Bangor, Gwynedd, LL57 2DG, UK

[3] Department of Biological and Environmental Sciences, University of Stirling, Stirling, FK9 4LA, Scotland, UK

Corresponding author

tmajor@bournemouth.ac.uk

Abstract

Understanding the success of animals in novel environments is increasingly important as human-mediated introductions continue to move species far beyond their natural ranges. Alongside these introductions, inhabited and agricultural areas are spreading, and correspondingly most animal introductions occur in populated areas. Commensal species which can live alongside humans by making use of specific conditions, structures, or prey, have a significant advantage. Introduced mammal species often use anthropogenic features in their environment and demonstrate a higher tolerance of human disturbance, but their importance remains understudied in ectotherms. The Aesculapian snake (*Zamenis longissimus*) is an ectotherm which has been introduced beyond the northern extremities of its natural range. To understand their persistence, we radio-tracked snakes daily over two active seasons, including high-frequency tracking of a subset of males. We investigated snake home range size using Autocorrelated Kernel Density Estimators (AKDE). Using AKDE-weighted Habitat Selection Functions we identified preferences for habitat features in a mosaic of habitats, and we used Integrated Step Selection Functions to further explore how these features influence movement. We revealed a particular preference for buildings in male snakes, while females preferred woodland. We demonstrate that the success of this ectothermic predator is likely tied to a willingness to use human features of the landscape.

Introduction

Through time, the areas inhabited by species are pushed and pulled in many directions by climate, habitat changes, interactions with other species, and human transport. The latter has become a particularly powerful force, introducing species to distant, novel habitats where they are exposed to a range of entirely different physical and climatic processes as well as different biotic interactions to those in their native ranges. Not all species are equally successful when faced with such pressures. Identifying the key characteristics underpinning the success or failure of species in novel environments can help predict winners and losers in a future where all animals face increased challenges (Moyle & Marchetti, 2006). Through the tracking of animal movements, we can understand traits which are key to success in unfamiliar locations, such as colonisation and dispersal abilities, and habitat requirements (Bubb, Lucas & Thom, 2002; Bartoszek et al., 2021; Kays et al., 2022). Using these insights, we can begin to explore how introduced species adapt to and impact their new habitats.

Many introduced species demonstrate a willingness to utilise human features, including fragmented landscapes and anthropogenic structures, to travel and hunt (Andersen et al., 2017), and have generally demonstrated greater tolerance of human disturbance than their native counterparts (Bielen et al., 2016). Using anthropogenic features is advantageous because non-native species are most likely to be introduced

in or near human-impacted habitats, and it is frequently generalist species that capitalise on these disturbed habitats and make good invaders (Marvier, Kareiva & Neubert, 2004). While much of the available literature focuses on terrestrial endotherms and their adaptability in the face of human dominated landscapes, comparatively little focuses on ectotherms – particularly snakes (Liu et al., 2020). This is despite several prominent examples of introduced snakes (albeit in more natural landscapes) having considerable impacts on the native fauna (Rodda & Savidge, 2007; Dorcas et al., 2012).

Snake introductions are not limited to warm climates. Aesculapian snakes (*Zamenis longissimus* (Laurenti, 1768)) are a constricting species in the family Colubridae. Native to mainland Europe, they range from France in the West to Iran in the East (Musilová, Zavadil & Kotlík, 2007). The species previously occupied a larger part of Northern Europe, only recently going extinct in Denmark during the early 1900s (Allentoft, Rasmussen & Kristensen, 2018), with remaining relict populations in Germany, Switzerland, the Czech Republic, and Poland. Aesculapian snakes have been introduced twice to the UK in modern times (Allentoft, Rasmussen & Kristensen, 2018). Snakes were introduced to Colwyn Bay, North Wales following an escape from the Welsh Mountain Zoo in the 1970s. This population represents the northernmost modern population of Aesculapian snakes, and its persistence here raises the question of how a species can expand northward, despite the constraints of ectothermy.

Over several decades, and especially since the advent of surgically implanted transmitters (Reinert & Cundall, 1982), radiotelemetry has become the backbone of snake spatial ecology. However, despite technological and methodological advancements, few studies have capitalised on recent advances in techniques and analyses to explore the full potential of telemetry data to illuminate the spatial ecology of reptiles. Much of the literature still uses outdated techniques and analyses to calculate habitat selection and home range size (Silva et al., 2020). Home range estimation measures such as minimum convex polygons and kernel density estimators have been continued to be used in determining the home range of animals (Silva et al., 2020). These methods assume independent and identically distributed data, and do not account for autocorrelation, where data points close in time are also close in space (de Solla, Bonduriansky & Brooks, 1999). This was generally acceptable for studies of animals which exhibit frequent movement, especially because of the large time lag between points that result from manually relocating the animal using VHF telemetry. However, for studies of animals which spend long periods inactive, such as snakes, autocorrelation presents a major concern and renders traditional home range estimators unreliable (Silva et al., 2020). Therefore we incorporated the novel approach of Autocorrelated Kernel Density Estimation (AKDE), which overcomes the limitations of traditional estimation techniques in that it accounts for autocorrelation, and these models present the additional benefit of providing confidence intervals for the estimates of home range size provided (Silva et al., 2022). Another continued limitation in most telemetry studies of reptiles involves short tracking durations and infrequent tracking

schedules, often ranging from once-daily to weekly tracking. Here, we included a two-hour daily tracking schedule in order to not only distinguish home range sizes more accurately but also to discern dispersal habitat selection. Using an advanced analysis called Integrated Step Selection Functions (ISSF) enabled the modelling of movement and habitat choice simultaneously.

The overall aim of this study was to investigate the spatial ecology of an introduced predator, adapted to a warmer climate but existing in cool North Wales. We used radiotelemetry to determine how these animals use their new range, and we set out to discover the home range and space use requirements of both male and female Aesculapian snakes. Our second goal was to discover the habitat preferences of this species, which is crucial to understanding its survival. As we had experienced snakes entering buildings and vegetation piles, we hypothesised that snakes may be reliant on human features of the landscape. Finally, we were keen to know the dispersal capabilities of this species. We wanted to learn which habitats represent pathways to mobile snakes, allowing us to infer their likely routes should this population spread into the surrounding area. We hypothesised that hedgerows, as linear features in the habitat, would represent pathways for snakes travelling longer distances.

Methods

Study Area and animals

The study site (approximately 1.72 km^2, see electronic supplementary material) is located between the town of Colwyn Bay and the village of Mochdre, North Wales, UK (53.28–53.29°N, -3.74–3.76°W). The area consists of a mosaic of habitats, including housing, with meadows and pastures separated by hedgerows. Most pastures are grazed periodically by sheep and cattle. The site also includes the Welsh Mountain Zoo covering an area of 0.15 km^2. The Zoo grounds are highly disturbed because of the constant maintenance involving the removal of trees or vegetation, landscaping or building of structures. There is also a patch of woodland in the north corner. Most of the animal faecal matter and vegetation waste is transported to a large dung heap in the southeastern corner of the zoo. Patches of deciduous forest and small patches of gorse scrub are scattered over the entire study site, and a small patch of woodland to the east of the Zoo was in the process of being removed for a new development in 2022. Roads are found throughout, with busy roads surrounding the zoo and the connecting meadows and pastures, with a dual carriageway (A55) at the site's northernmost extremity.

We implanted 21 adult Aesculapian snakes with radio transmitters during June – October 2021 and May – September 2022 (see supplementary material for tracking and capture summary). Our sample consisted of

13 males and eight females. Snakes were caught by hand, either during dedicated surveys, opportunistically, during radio-tracking activities, or following notification by members of the public. Two tracked individuals were caught by keepers at the Welsh Mountain Zoo during their daily activities. Because of the difficulty of finding Aesculapian snakes, which in this population takes approximately eight hours of searching per adult snake found, we radio-tracked any available adult snakes with sufficient body diameter to carry a transmitter. Snakes were transported to Bangor University for transmitter application, and we collected morphometric data including snout-vent length (SVL), tail length (TL) and mass (see supplementary material). We attempted to minimise the time snakes were kept in captivity for implantation procedures (n = 21 implantations, mean = 7.34 ± 6.7 days held, range = 1 – 23 days). Snakes were held in 70L plastic boxes (710 x 545 x 190 mm) in a temperature-controlled room at 21°C with suitable refuge and water provided *ad libitum*.

Depending on their size, snakes were implanted with 1.2, 1.4 or 1.6 g Holohil BD-2T radio transmitters (Holohil Inc, Canada) following Reinert & Cundall (1982), using isoflurane anaesthetic and butorphanol analgesia, with an internal securing stitch (Alworth, Hernandez & Divers, 2011). Post-implantation, snakes were kept overnight at a constant 21°C and released the following day. One snake (F159) was held for an additional day post-surgery to ensure wound closure. Snakes were released at their exact point of capture in dry conditions warmer than 14°C. The only exception was M180 who was caught basking on top of a hedge in a residential garden. The homeowner requested we release the snake a short distance from their garden, and we released him approximately 30 m away in a hedgerow. As snakes appeared to be behaving normally immediately after release, we began tracking the following day and did not discard any data.

Radiotelemetry

We employed two different tracking regimes. In order to assess longer distance movements and home range sizes, snakes were located twice daily as part of two daily tracking rounds, the first beginning at 10:00 and a second at 14:00. As we were a small team tracking many snakes, the timings of the tracks were not precise, but animals were usually tracked once in the morning and once in the afternoon, and almost always twice daily. See supplementary material for tracking periods and time lags.

In 2022 a subset of seven male snakes were located five times daily in order to determine what type of habitat the snakes use when they are moving, with the greater tracking frequency allowing us to capture more points in the movement pathway. These snakes were tracked in sessions beginning at 0900, 1100, 1300, 1500 and 1700. One female (F203) was tracked five times daily between 14/05/2022 and 14/06/2022, before switching to twice daily. After 20/08/2022, we began tracking the four male snakes with remaining transmitter battery twice daily (see electronic supplementary material). When we located a

snake, we recorded the temperature, humidity, and location, and noted any behaviour. We used a Garmin GPSMAP 64S to collect GPS locations. To avoid disturbing the snake and thus influencing their behaviour, we attempted to keep 10 metres between observers and tracked snakes, but within the confines of narrow gardens this was not always possible.

Snake home range estimation

To implement Autocorrelated Kernel Density Estimations (AKDE), we used the *ctmm* package (Calabrese, Fleming & Gurarie, 2016) and R v4.2.1 (R Core Team, 2022, p. 4) to fit continuous time stochastic process movement models to our snake movement data. We first checked individuals for range residency to ensure their range is no longer expanding by calculating the semi-variance function and visualising it using variogram analysis (Fleming & Calabrese, 2021). We removed individuals that did not demonstrate range-residency from the home range analysis. We fitted multiple movement models and used AICc to identify the model best fitting the autocorrelation structure for each snake. These were either the Ornstein-Uhlenbeck (OU) model where the animal exhibits Brownian motion restricted to a finite home range, or the OUF model with continuous-velocity motion restricted to a finite home range, or Independent Identically Distributed (IID). These prototype models are either isotropic, where there is equal diffusion in every direction, or anisotropic, where diffusion varies depending on direction (Calabrese, Fleming & Gurarie, 2016). For optimal performance we estimated autocorrelation and covariance bias using pertubative hybrid residual maximum likelihood (pHREML), accounting for both small absolute sample size and small effective sample size (Fleming et al., 2019). Absolute sample size refers to the total number of times the animal was located during the tracking period, while effective sample size is the entire tracking duration divided by how long it takes, on average, for the animal to cross its linear home range. We then fit AKDEs using the guidance provided by Silva et al., (2022). We used weighted AKDEc and pHREML to estimate home range size, which reduces oversmoothing of range limits, particularly in cases with small effective sample sizes (Silva et al., 2022). It also helps to address irregular sampling. Silva et al. (2022) recommend pHREML and AKDEc for effective sample sizes (range crossings) below 20, which applied to most individuals. Parametric bootstrapping can also be used to reduce estimation error, and we used ctmm.boot in the *ctmm* package to apply parametric bootstrapping to individuals with low effective sample sizes (Silva et al., 2018; Thompson et al., 2021).

Space use

In recent years there has been a rise in the use of dynamic Brownian Bridge Movement Models (dBBMMs) to estimate the home ranges of animals, but these estimators are not suitable for this purpose (Alston et al., 2022b). As these models are occurrence distribution estimators and not range distribution

estimators, these models use animal locations over time to interpolate where animals might have been during a tracking period, rather than extrapolating to their entire range (Alston et al., 2022b). However, dBBMMs have utitlity in estimating space use during the study period, and allow for comparison with other studies, giving a valuable impression of snake spatial ecology during the study period. As many snakes were unlikely to have sufficient tracking durations to facilitate home range estimation, we use estimates of space use derived from dBBMMs.

We estimated snake space use using the R package *move* v4.1.10 (Kranstaber, Smolla & Scharf, 2022). As snakes were generally active for a few days before spending between a few days and a week inactive, we specified a moving window of 11. With our twice daily tracking regime, this allowed us to detect variations in the behavioural state of snakes over a six day period (D'souza et al., 2021). We chose a margin size of three to allow detection of active vs inactive states (D'souza et al., 2021), and used the mean GPS error of our snake locations as the telemetry error. We used two daily tracks to model space use across all individuals.

Habitat selection

All analyses were conducted using R v4.2.1 (R Core Team, 2022). For all habitat use analyses, we used custom shape files of habitat types created in QGIS Using modified code from Smith et al (Smith et al., 2021) and Hodges et al (Hodges et al., 2022), we converted our raster layers into layers with continuous gradients denoting the Euclidean distance to habitat features. These layers were then inverted to avoid zero-inflation and to ensure that the resulting outputs were easy to interpret, as positive values indicate selection.

Integrated Resource (Habitat) Selection Functions

Traditional resource selection function analyses, now termed habitat selection functions (Fieberg et al., 2021), do not allow for autocorrelated data and assume independence between each point at which an animal is located. However, for high-resolution GPS location frequencies, or for animals which move infrequently, points are not independent from each other. To avoid the need to thin data to ensure independence of points, we utilised habitat selection functions informed by our AKDEs, which down-weight autocorrelated points rather than discarding them (Alston et al., 2022a). We used rsf.fit within the *ctmm* package (Fleming & Calabrese, 2021) to fit integrated resource selection functions to our snake tracking data, with simultaneously estimated spatial constraints. We used the Monte Carlo numerical integrator for likelihood evaluation, with a numerical error threshold of 0.05 for the parameter estimates and log-likelihood.

Integrated Step Selection Functions

We used Integrated Step Selection Functions (ISSF) to analyse how habitat types influenced the movements of Aesculapian snakes at the population scale. This allowed us to incorporate the movements of all tracked individuals, even those with short tracking durations that did not demonstrate range-residency. We split the data into male and female snakes and adapted code from Smith et al (2021) and Muff et al. (2019) to run mixed conditional Poisson regression models essentially operating as "population-level ISSFs" on our twice-daily tracking data. The first and third daily tracks of snakes which had been tracked five times daily were used to ensure comparability with our twice daily data. Using the *INLA* package v22.05.07 (Rue, Martino & Chopin, 2009) we ran a population-level ISSF to ascertain either association or avoidance of our eight habitat classifications. We removed zero distance steps (non-moves) from this analysis. We generated 200 random points per move, for step length we used a Gamma distribution and for turn angle Von Mises (Smith et al., 2021; Hodges et al., 2022), to facilitate comparison between locations the snakes selected and locations they did not. We created eight single-factor models, one for each habitat type, and all models included the interaction of turn angle and step length. As per Muff et al. (2019) the stratum-specific random effect of step was set to 0.0001. In keeping with Smith et al. (2021), we utilised a Penalised Complexity prior, PC (1, 0.05) for the random slope, which was individual, and uninformative normal priors were used for the fixed effects. Using the *INLA* package v22.05.07 (Rue, Martino & Chopin, 2009), we used nested Laplace approximations in fitting these models.

For the male snakes tracked five times daily, we ran both individual ISSFs as well as the "population-level ISSFs" described above to investigate whether Aesculapian snakes demonstrated attraction to or avoidance of habitat types at the scale of the whole population. For individual selection, we created ISSFs using *INLA* package v22.05.07 (Rue, Martino & Chopin, 2009), filtering steps using a resample rate of two hours with a tolerance of four hours to avoid bias from unexpected overnight movements. We created nine single-factor models, one for each habitat type, and one representing the null model. The individual habitat models also included the interaction of turn angle and step length, while the null model only included the interaction of step length and turn angle. Otherwise, we used the same settings as described above. We used AIC scores to determine which features most strongly influenced the habitat selection of Aesculapian snake individual movements from our ISSF. Models with the lowest AIC score or scores < 2 greater than the lowest were considered to have the largest influence.

Seasonality and shelter locations

We used two measures to determine seasonal behaviour changes in Aesculapian snakes, mean daily displacement and dBBMM-derived motion variance (Hodges et al., 2022). We use dBBMMs to visualise the space use of tracked snakes over the tracking period and ascertain areas of high use and re-use that are of particular significance to Aesculapian snakes. We also use the estimates of motion variance they provide to determine periods of heightened activity during the tracking period (Hodges et al., 2022).

Although mean daily displacement (MDD) has limitations, we were unable to estimate speed or distance travelled using continuous-time movement models [10], likely due to the relative infrequency of our data points compared to high-resolution GPS data. Despite the shortcomings of MDD (Rowcliffe et al., 2012), we kept to a strict tracking schedule of at least two tracks per day for all individuals, allowing increased confidence in the MDD estimates (see electronic supplementary material). To investigate seasonality, we filtered the first and third daily tracks of snakes which received five tracks per day to allow comparison between all individuals. We summed the movement distance across these two daily tracks to create our values for daily displacement. We opportunistically collected observational data on the breeding behaviour of this population during tracking activities, which allowed us to better inform the dates of the mating and egg-laying seasons.

To understand areas snakes used for long periods, we first created move objects in R using twice daily tracking data for all 21 individuals with the package *move* v4.1.10 (Kranstaber, Smolla & Scharf, 2022). Using the *recurse* package v1.1.2 (Bracis, Bildstein & Mueller, 2018), we set a radius of five meters around each location a snake visited. We recorded the amount of time each snake spent in the same location for multiple fixes as time spent stationary. Finally, we used the recurse package to visualise places the snakes had spent long periods, and recorded what these locations were by overlaying the GPS locations on a map. We verified the locations using our behavioural notes.

Limitations and data statement

In line with the STRANGE framework (Webster & Rutz, 2020), we recognise that there may be bias in the trappability and self-selection of our sample of snakes. Snakes were frequently near people or their dwellings when captured. Seven of the 13 male snakes were captured at different times under one tarpaulin covering a wood pile in the garden of a residential home, while three of eight females came from one garden containing multiple mature compost heaps. As many snakes were found near each other, there is also a higher likelihood they are related.

All Figs were created using R v4.2.1 (https://r-project.org/). For data manipulation we used the *amt* package v0.1.7 (Signer, Fieberg & Avgar, 2019) and the *dplyr* package v1.0.10 (Wickham et al., 2022). For data visualisation we used *ggplot2* v3.3.6 (Wickham, 2016). For visualising movement data and

creating tracks we used *move* v4.1.10 (Kranstaber, Smolla & Scharf, 2022). All data and code used in this study are available from the online repository figshare (link to be added).

Results

Movements

We tracked 13 adult male and eight adult female Aesculapian snakes between June and October 2021 and May and September 2022. The average tracking duration was 56.67 ± SE 7.85 days (range 4 – 126 days). We collected a total of 3232 snake locations, which included 947 relocations (a move from the previous location). The mean daily displacement (MDD) for six male snakes on the twice daily tracking regime was 38.45 ± 21.33 m, and for the seven males on the five times daily regime it was 52.34 ± 26.43 m. The MDD of eight females tracked twice daily was 26.14 ± 18.55 m.

Snake home ranges

Eight males and three females were found to have stable home ranges using variogram analysis (see electronic supplementary material). The remaining ten individuals did not reach an aysmptote (Fleming et al., 2014). For these individuals, the short tracking durations meant that there was insufficient information to inform our home range estimation model, and they were excluded from Autocorrelated Kernel Density Estimation (AKDE). Full model results for all individuals can be found in Table 1. The mean effective sample size for snakes included in the AKDE analysis was 12.92 ± 8.57 (range 4.29 – 35.57). These Figs are relatively low and demonstrate that using the pHREML method and weighting the AKDEs was justified (Silva et al., 2022). The mean 95% AKDE estimate for home range size for the three range resident females was 23.32 ± 29.74 ha (range 0.28 – 65.31 ha). For eight range resident males it was 28.86 ± 28.42 ha (range 2.05 – 92.16 ha). For range resident snakes (Fig 1), the top models were Ornstein-Uhlenbeck (OU) or Ornstein-Uhlenbeck foraging (OUF). Two individuals, F159 and M218, had low effective sample sizes of six and 4.3 respectively, with pHREML bias in the order of 3% or higher (Table 1), and we applied parametric bootstrapping to these individuals. All models reflected elliptical home ranges (anisotropic) except for F158 which was more circular (isotropic). The traditional KDE approach of using Independent Identically Distributed (IID) points proved ineffective and the IID models had high dAICc values (see electronic supplementary material).

Table 1: Results of the snake AKDE home range analysis. Effective sample size refers to the number of times the animal crossed its home range during the tracking period, while absolute sample size is the total number of observations (fixes). Contour area estimates of home range for individuals presented in

hectares. F159 and M218 had parametric bootstrapping applied to reduce error on their home-range estimates and the estimates displayed are post-bootstrapping.

ID	AICc top model	Effective sample size	Absolute sample size	95% AKDE lower CI (ha)	95% AKDE estimate (ha)	95% AKDE upper CI (ha)	pHREML bias	Parametric bootstrap bias
F158	OU	8.72	102	1.97	4.37	7.72	0.013	-
F159	OUF anisotropic	4.71	102	20.10	64.75	135.03	0.028	0.0096
F177	OU anisotropic	35.57	126	0.19	0.28	0.38	0.001	-
M031	OU anisotropic	19.44	165	1.24	2.05	3.05	0.003	-
M137	OU anisotropic	9.36	209	9.98	21.44	37.20	0.011	-
M139	OU anisotropic	8.69	167	7.83	17.41	30.75	0.013	-
M154	OU anisotropic	7.66	302	4.54	10.75	19.59	0.017	-
M180	OU anisotropic	14.89	96	1.46	2.61	4.10	0.005	-
M202	OU anisotropic	8.83	457	23.37	51.57	90.74	0.013	-
M209	OU anisotropic	18.74	423	19.72	32.89	49.37	0.003	-
M218	OU anisotropic	4.08	192	100	120.04	261.50	0.054	0.0147

Fig 1. AKDE home ranges for tracked snakes that demonstrated range-residency. A-D) Male snakes. E, F) Female snakes. Darkest shading in the centre represents the lower confidence interval for the 95% home range contour, medium shading is the 95% contour, and the lightest shading is the upper confidence interval. Points represent location data of each animal. While F203 did not demonstrate range residency, we plot the data here for illustration purposes as she was tracked for a relatively long period of 65 days.

Snake space use

We estimated space use using dBBMMs (Table 2). We excluded three snakes with tracking durations less than 14 days (F212, F219, and M073) from the following means as their space use estimates would likely skew the results. For females, the mean area of the 95% confidence occurrence distributions was 2.09 ± 3.79 ha (range = 0.02 – 10.51 ha). The mean area of the 95% confidence occurrence distributions for males was 6.34 ± 7.10 ha (range 0.46 – 20.72 ha).

Table 2. dBBMM occurrence distributions and mean daily displacement (MDD) for the Aesculapian snakes tracked in this study.

ID	90% (ha)	95% (ha)	99% (ha)	Tracking duration (days)	Total distance moved (m)	MDD (m)	Maximum daily distance (m)
F050	0.02	0.05	0.16	28	216.20	7.74	56.78
F142	0.02	0.02	0.04	33	264.26	8.05	58.09
F158	0.55	1.30	3.23	50	1028.29	20.57	92.10
F159	5.91	10.51	18.42	61	3673.04	60.38	364.43
F177	0.21	0.32	0.59	90	782.51	8.68	44.67
F203	0.19	0.32	0.92	65	1282.96	19.70	72.06
F212	0.07	0.09	0.13	4	157.93	38.04	64.73
F219	0.01	0.01	0.02	12	555.29	46.00	244.47
M031	0.63	1.03	1.97	33	1135.56	34.20	129.11
M073	9.03	12.48	20.82	9	698.28	78.71	206.39
M074	0.01	0.01	0.02	30	179.99	5.97	64.96
M137	1.82	4.51	14.49	126	3894.85	30.95	382.88
M139	2.27	7.21	22.72	95	3903.90	41.10	559.30
M149	1.56	2.76	6.41	37	1706.27	46.37	336.90
M154	1.79	3.67	7.57	74	2427.55	32.70	107.16
M178	0.29	0.46	0.87	83	999.82	12.04	255.31
M180	1.31	2.13	4.48	57	1225.27	21.54	125.12
M202	5.55	10.75	25.43	112	7935.06	70.85	566.25
M209	14.10	21.00	35.59	107	7387.04	69.03	365.16
M217	0.35	1.85	5.27	17	1477.92	86.94	383.95
M218	11.94	20.72	43.71	67	4469.91	66.73	586.54

Individual habitat selection via AKDE-weighted RSF

We had sufficient data to inform range-residency and perform the habitat selection analysis on three female and eight male Aesculapian snakes. These parameter estimates for resource selection are visualised in Fig 2. The results for the remaining snakes can be found in the supplementary material. In male snakes, buildings were the most commonly selected-for habitat type with five of eight individuals demonstrating positive selection for buildings and two more strongly suggesting it without definitive evidence (Fig 2). M137 and M139 showed a preference for woodland, while M202 and M209 were associated with pasture.

One chose gardens (M180), one selected meadows (M202), and similarly only (M154) showed a preference for hedgerows. For females, F159 showed avoidance of pasture. We were unable to determine individual preference or for any habitat type for females in this analysis. With regards to males avoiding habitat types, three individuals (M031, M180 and M202) avoided roads, while M202 avoided woodland.

Fig 2. Coefficients from the weighted AKDE habitat selection functions for range resident snakes. Each plot displays the habitat selection of one individual snake. Positive values indicate selection for a habitat type, while negative values indicate avoidance. Error bars represent 95% confidence intervals.

ISSF at the population scale

The ISSF analysis suggests woodland is of importance for females at the population-level (Fig 3). Males demonstrated habitat generalism, showing selection for meadows, scrub, road surfaces, and possibly hedgerows, but appearing to select areas nearer to buildings and gardens most strongly (Fig 3). We were unable to detect differences in step length associated with different habitat types for either females or males at the population scale (see electronic supplementary material).

Fig 3. Results of the ISSF analysis at the population-level for female snakes (n = 8) and male snakes (n = 13). Bars represent 95% confidence intervals. Positive values indicate selection toward a particular habitat type, while negative values indicate avoidance.

Step length from five times daily tracking

We included the data from six of the seven males that were tracked five times daily, excluding M074 who spent 29 of 30 tracking days stationary. We investigated the influence of habitat type on the step lengths of individual snakes (Fig 4). For M202, shorter steps were associated with buildings and roads. For M218, both proximity to pasture and hedgerows were associated with shorter steps.

We also determined which features influenced the habitat selection of snakes. The top model was pasture for M218. Gardens were top or second for M154 and M217. Hedgerows were top or second for M154 and M209, while M207. M031, M154, and M202 were influenced by buildings (see electronic supplementary material). Model selection results do not necessarily equate to definite selection or avoidance, however. At the individual level, two snakes, M154 and M202, preferred to be closer to meadows and pasture. M218 showed a positive association with hedgerows and scrub. None of the remaining males tracked five times

daily showed definitive habitat association in this analysis. We also ran the population-level analysis for this group, who demonstrated a preference for hedgerows, buildings and scrub, but we found no significant influence of habitat type on step length using the population-level analysis with this group (see electronic supplementary material).

Fig 4. Individual integrated step selection functions demonstrating the interaction between step length and habitat types for Aesculapian snakes under the five times daily tracking regime. Positive values indicate longer steps associated with a habitat feature, and negative values indicate shorter steps. Error bars indicate 95% confidence intervals. Circles indicate the features which were included in models with the lowest AIC score or scores less than two higher.

Seasonality

Female movement showed a visible peak in motion variance during the egg-laying season from July to mid August (Fig 5). The mean daily displacement (MDD) of females during the egg-laying season was 34.05 ± 75.45 m/day compared to 12.52 ± 32.33 m/day during the rest of the year (Fig 6). Across all females there was only one day where an individual moved > 100 m outside of the egg-laying season, and females were sedentary outside of this period. We discovered eggs inside the compost heap of a residential property immediately after F177 left it on 10/08/2021. F159 made an uncharacteristically long 364m move in mid-July that we interpret as nest searching, before spending three days (13/07/2021 - 16/07/2021) inside the dung pile within the Welsh Mountain Zoo. While we could not locate her eggs despite extensive search, this may be due to the size of the pile, which is approximately 10 m across. We found a separate clutch of eggs inside this pile in 2019, confirming that it is an egg-laying site for the species. One further Aesculapian snake egg was found by Zoo staff in a pile of wood chippings 20 m from the dung heap on the Zoo site in 2021.

Fig 5. Motion variance plot for females and males through the study periods in 2021 and 2022. Peaks represent increased movement distances.

Fig 6. Raincloud plot visualising the mean daily displacement of eight tracked female Aesculapian snakes and 13 tracked male Aesculapian snakes through 2021-2022. Each point represents the distance moved by a snake on an individual day. Non-moves are excluded from the box and density plots, with

numbers inside the box showing the number of moving days in each season. Days without movement are plotted to the far left along with a count. Mean daily displacement for each season, with non-moves included, are printed along with a small black arrow. The egg-laying season (July 1st – August 16th) and the mating season (15th May – 30th June) are compared with the rest of the active season for females and males respectively. Fig created using code adapted from (Hodges et al., 2022).

The males showed a visible peak in motion variance the mating season between mid May and the end of June (Fig 5). The MDD of males during the mating season was 68.7 ± 112.22 m/day compared to 26.3 ± 58.39 m/day during the rest of the year (Fig 6). Males only moved distances > 500 m per day during the mating season, despite occasional periods of high activity in mid-summer. We observed one tracked male mating on 13/06/2021 and another on 24/05/2022. We witnessed one of our tracked males in combat with an unknown male on 15/06/2022. These observations reinforce our understanding of the timings of the mating season of this species in Wales.

Snake mortality

Of our sample of 21 tracked snakes, five died during their tracking periods, and one died after tracking was completed. In total, three females died. F177 was tracked during 2021 until the transmitter ran out of battery, and was then found dead the following year on 15/08/2022 after a car strike (see supplementary material). F142 was also killed by a car strike on 07/07/2021 after being tracked for 33 days, on the same two-laned stretch of road as F177. Both snakes were gravid at the time of death, containing four and seven well-developed eggs respectively. Lastly, F159 was cannibalised by a tracked male (M137) in August 2021 (Major et al., 2023).

Three of 13 male snakes died during the study. In 2021, M073 was predated by buzzards, with his transmitter signal discovered in a pine tree containing a buzzard nest, approximately 30 m high up. M149 died from mammal predation. The snake was found at the edge of a meadow with approximately one metre of trampled grass in all directions with a broken neck. Small sections of the snake had been consumed. We suspect either a stoat, badger, or domestic cat was responsible. Finally, in the 2022 season, M031 was killed by a car strike on the entrance road within the Welsh Mountain Zoo grounds.

Time spent sheltering

All snakes spent time stationary, being located at the same location for at least two tracks in succession. The mean time spent stationary of all females was 4.91 ± 4.04 days (0.5 - 14.27 days). For all males, the mean time spent stationary was 5.14 ± 6.67 days (range 0.82 - 27.61 days). Generally, when snakes were in the same location for multiple successive fixes, they were inside shelter. The exception were two snakes

who were repeatedly found in basking sites in vegetation at the edges of roads (M137 and M218). Females selected a road verge (n = 1), compost or vegetation piles (n = 4), and buildings (n = 3) as repeated or long-term shelter. Males chose a road verge (n = 1), compost or vegetation pile (n = 1), or buildings (n = 10). Five males used two different buildings for shelter, while three males used three different buildings. These shelter sites were often used for long periods, and seven males and seven females spent ≥ 10 days in an individual shelter (see supplementary material).

Discussion

Our suite of habitat and step selection analyses reveal that male Aesculapian snakes show a distinct preference for buildings in their introduced range in North Wales, with seven of eight individuals in our AKDE-weighted habitat selection function selecting buildings as habitat, and our population SSF model highlighted their importance to male snakes. Like many introduced species, Aesculapian snakes were introduced close to an urban area following escape from captivity. Urban areas often contain under-utilised resources, and introduced species which can capitalise on them have a significant advantage (Mitchell, Folt & Hall, 2021). For snakes, anthropogenic structures such as buildings and culverts provide shelter, thermoregulatory opportunities and egg-laying sites (Keller & Heske, 2000; Lelièvre et al., 2010; Hanslowe et al., 2016; Hodges et al., 2022; Yu et al., 2022). Snakes frequently took shelter in human features of the habitat, and compost heaps, vegetation piles, and buildings represented long-term shelter for female snakes. Females spent long periods inactive during the Spring and early summer, remaining in shelter - particularly compost heaps. Males frequently used buildings as shelter sites, with one individual using a hole in a road verge and one a vegetation pile as long-term or revisited shelter sites.

We observed Aesculapian snakes actively seeking and returning to use inhabited buildings and were observed climbing large structures to access the attics and wall cavities of houses. This attraction towards anthropogenic features is unusual behaviour compared with native snake species in the UK that often avoid built up urban areas. The Adder (*Vipera berus*) and smooth snake (*Coronella austriaca*) are rarely found in human-dominated environments. Grass snakes (Natrix sp. including *Natrix helvetica*) can be found in anthropogenic environments and will use compost heaps, manmade structures, or ponds in gardens, but their use of anthropogenic features is significantly less extensive (Wisler, Hofer & Arlettaz, 2008; Reading and Jofré, 2009; Löwenborg et al., 2010). With urbanisation continuing unabated, the ability to survive in human-dominated landscapes is valuable to individual species and vital for maintaining biodiversity (Collins, Magle & Gallo, 2021). Herpetofauna and invertebrates remain understudied in this realm, and our suite of analytical approaches represents an unusually detailed investigation into the lives of a cryptic reptile species.

Our evidence here suggests that, like other snakes, Aesculapian snakes have peaks in activity due to their reproductive cycles (Row, Blouin-Demers & Lougheed, 2012; Marshall et al., 2020). The activity of male snakes peaked in the mating season in May and June, coinciding with observations of male-male combat and mating, while females exhibited a definite peak during summer when they travel to lay their eggs. We discovered eggs in rotting vegetation built up by humans - a compost heap, a pile of wood chippings, and a dung heap at the Welsh Mountain Zoo, further demonstrating the importance of human elements of the habitat to this introduced species.

Despite their ability to benefit from human features of the landscape, the semi-rural range of this introduced predator also contains dangers. Three of 21 tracked snakes died to road mortality. Road mortalities can be high for juvenile Aesculapian snakes (Kovar et al., 2014), and our study site lacks any culverts beneath the roads, which are known to be utilised by snakes for road crossing (Jones et al., 2022). Indeed, road mortality was found to be low for adult Aesculapian snakes in a population where culverts allowed safe passage under roads (Kovar et al., 2014). Two females died on roads while heavily gravid, potentially suggesting an increased risk of road mortality when travelling to lay eggs. Reptiles are known to increase their movement distances in highly disturbed areas, despite keeping smaller home ranges (Doherty, Hays & Driscoll, 2021), further exacerbating the risk of road mortality. Three snakes demonstrated avoidance of roads in this study, suggesting they may be responsible for limiting the expansion of this species, although this is more likely the case for wider roads (Roe, Gibson & Kingsbury, 2006; Bauder et al., 2021). Two other mortalities in this study were caused by a mammalian predator and a buzzard, and one was cannibalised by an Aesculapian snake (Major et al., 2023).

Conclusion

In summary, this study demonstrates the importance of human-dominated habitats to an introduced predator. While Aesculapian snakes are present in the fossil record of the UK, they have been absent for likely 300,000 years (Ashton et al., 1994). Worldwide, animal ranges are shifting poleward or to higher elevation as the climate warms dramatically because of human activity (Lenoir & Svenning, 2015). The UK is now home to an increasing number of mobile species which can travel over sea from further South, including numerous moths and butterflies (Sparks et al., 2007), and wetland birds (Hiley et al., 2013). Aesculapian snakes have similarly migrated northward, only via human transport instead of natural means. That said, it seems likely that North Wales represents the northernmost tolerance limit of this species in current climatic conditions. The use of buildings for shelter and vegetation piles for egg laying appear to be important to their success in a temperate climate that is further north than any remaining native populations. However, simply being successful in an area is not evidence to suggest the area contains ideal conditions (Hawley Matlaga et al., 2021), and the broad range of habitats selected by individual snakes in

this study suggest that Aesculapian snakes are adaptable generalists, capable of using mixed habitat and unafraid of using buildings and other features in close proximity to humans.

Acknowledgements

We are indebted to the staff of the Welsh Mountain Zoo for their tireless support and assistance. Thank you also to the generous residents of Colwyn Bay who allowed us to conduct fieldwork on their properties with enthusiasm. We thank Henry Crisp, Jack Pearce, Gem Shinasi, and Andrea Pozzi for volunteering their time to radio-track snakes. Thank you to Becca Snell for assisting with radio transmitter implantations.

References

Allentoft ME, Rasmussen AR, Kristensen HV. 2018. Centuries-old DNA from an extinct population of aesculapian snake (zamenis longissimus) offers new phylogeographic insight. *Diversity* 10. DOI: 10.3390/D10010014.

Alston JM, Fleming CH, Kays R, Streicher JP, Downs CT, Ramesh T, Reineking B, Calabrese JM. 2022a. Mitigating pseudoreplication and bias in resource selection functions with autocorrelation-informed weighting. *Methods in Ecology and Evolution*:2041–210X.14025. DOI: 10.1111/2041-210X.14025.

Alston JM, Fleming CH, Noonan MJ, Tucker MA, Silva I, Folta C, Akre TSB, Ali AH, Belant JL, Beyer D, Blaum N, Böhning-Gaese K, de Paula RC, Dekker J, Drescher-Lehman J, Farwig N, Fichtel C, Fischer C, Ford AT, Janssen R, Jeltsch F, Kappeler PM, LaPoint SD, Markham AC, Medici EP, Morato RG, Nathan R, Olson KA, Patterson BD, Petroelje TR, Ramalho EE, Rösner S, Oliveira Santos LG, Schabo DG, Selva N, Sergiel A, Spiegel O, Ullmann W, Zieba F, Zwijacz-Kozica T, Wittemyer G, Fagan WF, Müller T, Calabrese JM. 2022b. Clarifying space use concepts in ecology: range vs. occurrence distributions. DOI: 10.1101/2022.09.29.509951.

Alworth LC, Hernandez SM, Divers SJ. 2011. Laboratory reptile surgery: principles and techniques. *Journal of the American Association for Laboratory Animal Science* 50:11–26.

Andersen GE, Johnson CN, Barmuta LA, Jones ME. 2017. Use of anthropogenic linear features by two medium-sized carnivores in reserved and agricultural landscapes. *Scientific Reports* 7:11624. DOI: 10.1038/s41598-017-11454-z.

Ashton NM, Bowen DQ, Holman JA, Hunt CO, Irving BG, Kemp RA, Lewis SG, McNabb J, Parfitt S, Seddon MB. 1994. Excavations at the Lower Palaeolithic site at East Farm, Barnham, Suffolk 1989-92. *Journal of the Geological Society* 151:599–605. DOI: 10.1144/gsjgs.151.4.0599.

Bartoszek IA, Smith BJ, Reed RN, Hart KM. 2021. Spatial ecology of invasive Burmese pythons in southwestern Florida. *Ecosphere* 12. DOI: 10.1002/ecs2.3564.

Bauder JM, Peterman WE, Spear SF, Jenkins CL, Whiteley AR, McGarigal K. 2021. Multiscale assessment of functional connectivity: Landscape genetics of eastern indigo snakes in an anthropogenically fragmented landscape in central Florida. *Molecular Ecology* 30:3422–3438. DOI: 10.1111/mec.15979.

Bielen A, Bošnjak I, Sepčić K, Jaklič M, Cvitanić M, Lušić J, Lajtner J, Simčič T, Hudina S. 2016. Differences in tolerance to anthropogenic stress between invasive and native bivalves. *Science of The Total Environment* 543:449–459. DOI: 10.1016/j.scitotenv.2015.11.049.

Bracis C, Bildstein KL, Mueller T. 2018. Revisitation analysis uncovers spatio-temporal patterns in animal movement data. *Ecography*:1–11. DOI: 10.1111/ecog.03618.

Bubb DH, Lucas MC, Thom TJ. 2002. Winter movements and activity of signal crayfish Pacifastacus leniusculus in an upland river, determined by radio telemetry. In: Thorstad EB, Fleming IA, Næsje TF eds. *Aquatic Telemetry*. Dordrecht: Springer Netherlands, 111–119. DOI: 10.1007/978-94-017-0771-8_13.

Calabrese JM, Fleming CH, Gurarie E. 2016. ctmm: An R package for analyzing animal relocation data as a continuous-time stochastic process. *Methods in Ecology and Evolution* 7:1124–1132. DOI: 10.1111/2041-210X.12559.

Collins MK, Magle SB, Gallo T. 2021. Global trends in urban wildlife ecology and conservation. *Biological Conservation* 261:109236. DOI: 10.1016/j.biocon.2021.109236.

Doherty TS, Hays GC, Driscoll DA. 2021. Human disturbance causes widespread disruption of animal movement. *Nature Ecology & Evolution* 5:513–519. DOI: 10.1038/s41559-020-01380-1.

Dorcas ME, Willson JD, Reed RN, Snow RW, Rochford MR, Miller MA, Meshaka WE, Andreadis PT, Mazzotti FJ, Romagosa CM, Hart KM. 2012. Severe mammal declines coincide with proliferation of invasive Burmese pythons in Everglades National Park. *Proceedings of the National Academy of Sciences* 109:2418–2422. DOI: 10.1073/pnas.1115226109.

D'souza A, Gale GA, Marshall BM, Khamcha D, Waengsothorn S, Strine CT. 2021. Space use and activity of Boiga cyanea – A major songbird nest predator in a seasonal tropical forest in Thailand. *Global Ecology and Conservation* 32:e01875. DOI: 10.1016/j.gecco.2021.e01875.

Fieberg J, Signer J, Smith B, Avgar T. 2021. A 'How to' guide for interpreting parameters in habitat-selection analyses. *Journal of Animal Ecology* 90:1027–1043. DOI: 10.1111/1365-2656.13441.

Fleming CH, Calabrese JM. 2021. ctmm: continuous-time movement modeling.

Fleming CH, Calabrese JM, Mueller T, Olson KA, Leimgruber P, Fagan WF. 2014. From Fine-Scale Foraging to Home Ranges: A Semivariance Approach to Identifying Movement Modes across Spatiotemporal Scales. *The American Naturalist* 183:E154–E167. DOI: 10.1086/675504.

Fleming CH, Noonan MJ, Medici EP, Calabrese JM. 2019. Overcoming the challenge of small effective sample sizes in home-range estimation. *Methods in Ecology and Evolution* 10:1679–1689. DOI: 10.1111/2041-210X.13270.

Hanslowe EB, Falk BG, Collier MAM, Josimovich JM, Rahill TA, Reed RN. 2016. First Record of Invasive Burmese Python Oviposition and Brooding Inside an Anthropogenic Structure. *Southeastern Naturalist* 15:103–106.

Hawley Matlaga TJ, Burrowes PA, Hernández-Pacheco R, Pena J, Sutherland C, Wood TE. 2021. Warming increases activity in the common tropical frog Eleutherodactylus coqui. *Climate Change Ecology* 2:100041. DOI: 10.1016/j.ecochg.2021.100041.

Hiley JR, Bradbury RB, Holling M, Thomas CD. 2013. Protected areas act as establishment centres for species colonizing the UK. *Proceedings of the Royal Society B: Biological Sciences* 280:20122310. DOI: 10.1098/rspb.2012.2310.

Hodges CW, Marshall BM, Iii JGH, Strine CT. 2022. Malayan kraits (Bungarus candidus) show affinity to anthropogenic structures in a human dominated landscape. *Scientific Reports* 12:7139.

Jones MD, Marshall BM, Smith SN, Crane M, Silva I, Artchawakom T, Suwanwaree P, Waengsothorn S, Wüster W, Goode M, Strine CT. 2022. How do King Cobras move across a major highway? Unintentional wildlife crossing structures may facilitate movement. *Ecology and Evolution* 12:e8691. DOI: 10.1002/ece3.8691.

Kays R, Davidson SC, Berger M, Bohrer G, Fiedler W, Flack A, Hirt J, Hahn C, Gauggel D, Russell B, Kölzsch A, Lohr A, Partecke J, Quetting M, Safi K, Scharf A, Schneider G, Lang I, Schaeuffelhut F, Landwehr M, Storhas M, van Schalkwyk L, Vinciguerra C, Weinzierl R, Wikelski M. 2022. The Movebank system for studying global animal movement and demography. *Methods in Ecology and Evolution* 13:419–431. DOI: 10.1111/2041-210X.13767.

Keller WL, Heske EJ. 2000. Habitat Use by Three Species of Snakes at the Middle Fork Fish and Wildlife Area, Illinois. *Journal of Herpetology* 34:558–564. DOI: 10.2307/1565271.

Kovar R, Brabec M, Vita R, Bocek R. 2014. Mortality Rate and Activity Patterns of an Aesculapian Snake (*Zamenis longissimus*) Population Divided by a Busy Road. *Journal of Herpetology* 48:24–33. DOI: 10.1670/12-090.

Kranstaber B, Smolla M, Scharf AK. 2022. move: Visualizing and Analyzing Animal Track Data.

Lelièvre H, Blouin-Demers G, Bonnet X, Lourdais O. 2010. Thermal benefits of artificial shelters in snakes: A radiotelemetric study of two sympatric colubrids. *Journal of Thermal Biology* 35:324–331. DOI: 10.1016/j.jtherbio.2010.06.011.

Lenoir J, Svenning J-C. 2015. Climate-related range shifts – a global multidimensional synthesis and new research directions. *Ecography* 38:15–28. DOI: 10.1111/ecog.00967.

Liu C, Wolter C, Xian W, Jeschke JM. 2020. Most invasive species largely conserve their climatic niche. *Proceedings of the National Academy of Sciences* 117:23643–23651. DOI: 10.1073/pnas.2004289117.

Major T, Bracegirdle R, Gandini A, Russell GL, Pozzi AV, Morgan R, Mulley JF, Wüster W. 2023. Mate today, gone tomorrow: male-on-female cannibalism in Zamenis longissimus (Laurenti, 1768) in North Wales. *Herpetology Notes* 16:51–54.

Marshall BM, Crane M, Silva I, Strine CT, Jones MD, Hodges CW, Suwanwaree P, Artchawakom T, Waengsothorn S, Goode M. 2020. No room to roam: King Cobras reduce movement in agriculture. *Movement Ecology* 8:1–14. DOI: 10.1186/s40462-020-00219-5.

Marvier M, Kareiva P, Neubert MG. 2004. Habitat Destruction, Fragmentation, and Disturbance Promote Invasion by Habitat Generalists in a Multispecies Metapopulation. *Risk Analysis* 24:869–878. DOI: 10.1111/j.0272-4332.2004.00485.x.

Mitchell TS, Folt B, Hall JM. 2021. Dumpsters and other anthropogenic structures as habitat for invasive African rock agama lizards in Florida. *Biological Invasions* 23:2689–2693. DOI: 10.1007/s10530-021-02537-0.

Moyle PB, Marchetti MP. 2006. Predicting Invasion Success: Freshwater Fishes in California as a Model. *BioScience* 56:515–524. DOI: 10.1641/0006-3568(2006)56[515:PISFFI]2.0.CO;2.

Muff S, Signer J, Fieberg J. 2019. Accounting for individual-specific variation in habitat-selection studies: Efficient estimation of mixed-effects models using Bayesian or frequentist computation. *Journal of Animal Ecology* 89:80–92. DOI: 10.1111/1365-2656.13087.

Musilová R, Zavadil V, Kotlík P. 2007. Isolated populations of Zamenis longissimus (Reptilia: Squamata) above the northern limit of the continuous range in Europe: origin and conservation status. *Acta Societatis Zoologicae Bohemicae* 71:197–208.

QGIS Development Team. 2023. QGIS Geographic Information System. Open Source Geospatial Foundation Project.

R Core Team. 2022. R: A language and environment for statistical computing. R Foundation for Statistical Computing, Vienna, Austria.

Reinert HK, Cundall D. 1982. An improved surgical implantation method for radio-tracking snakes. *Copeia* 1982:702–705. DOI: 10.2307/1444674.

Rickart EA, Balete DS, Rowe RJ, Heaney LR. 2011. Mammals of the northern Philippines: tolerance for habitat disturbance and resistance to invasive species in an endemic insular fauna. *Diversity and Distributions* 17:530–541. DOI: 10.1111/j.1472-4642.2011.00758.x.

Rodda GH, Savidge J a. 2007. Biology and Impacts of Pacific Island Invasive Species. 2. Boiga irregularis, the Brown Tree Snake (Reptilia: Colubridae)1. *Pacific Science* 61:307–324. DOI: 10.2984/1534-6188(2007)61[307:BAIOPI]2.0.CO;2.

Roe JH, Gibson J, Kingsbury BA. 2006. Beyond the wetland border: Estimating the impact of roads for two species of water snakes. *Biological Conservation* 130:161–168. DOI: 10.1016/j.biocon.2005.12.010.

Row JR, Blouin-Demers G, Lougheed SC. 2012. Movements and habitat use of Eastern Foxsnakes (Pantherophis gloydi) in two areas varying in size and fragmentation. *Journal of Herpetology* 46:94–99. DOI: 10.1670/10-262.

Rowcliffe JM, Carbone C, Kays R, Kranstauber B, Jansen PA. 2012. Bias in estimating animal travel distance: the effect of sampling frequency. *Methods in Ecology and Evolution* 3:653–662. DOI: 10.1111/j.2041-210X.2012.00197.x.

Rue H, Martino S, Chopin N. 2009. Approximate Bayesian Inference for Latent Gaussian models by using Integrated Nested Laplace Approximations. *Journal of the Royal Statistical Society Series B: Statistical Methodology* 71:319–392. DOI: 10.1111/j.1467-9868.2008.00700.x.

Signer J, Fieberg J, Avgar T. 2019. Animal movement tools (amt): R package for managing tracking data and conducting habitat selection analyses. *Ecology and Evolution* 9:880–890. DOI: 10.1002/ece3.4823.

Silva I, Crane M, Marshall BM, Strine CT. 2020. Reptiles on the wrong track? Moving beyond traditional estimators with dynamic Brownian Bridge Movement Models. *Movement Ecology* 8:1–13. DOI: 10.1186/s40462-020-00229-3.

Silva I, Crane M, Suwanwaree P, Strine C, Goode M. 2018. Using dynamic brownian bridge movement models to identify home range size and movement patterns in king cobras. *PLoS ONE* 13:1–20. DOI: 10.1371/journal.pone.0203449.

Silva I, Fleming CH, Noonan MJ, Alston J, Folta C, Fagan WF, Calabrese JM. 2022. Autocorrelation-informed home range estimation: A review and practical guide. *Methods in Ecology and Evolution* 13:534–544. DOI: 10.1111/2041-210X.13786.

Smith SN, Jones MD, Marshall BM, Waengsothorn S, Gale GA, Strine CT. 2021. Native Burmese pythons exhibit site fidelity and preference for aquatic habitats in an agricultural mosaic. *Scientific Reports* 11:1–13. DOI: 10.1038/s41598-021-86640-1.

de Solla SR, Bonduriansky R, Brooks RJ. 1999. Eliminating autocorrelation reduces biological relevance of home range estimates. *Journal of Animal Ecology* 68:221–234. DOI: 10.1046/j.1365-2656.1999.00279.x.

Sparks TH, Dennis RLH, Croxton PJ, Cade M. 2007. Increased migration of Lepidoptera linked to climate change. *European Journal of Entomology* 104:139–143. DOI: 10.14411/eje.2007.019.

Thompson JJ, Morato RG, Niebuhr BB, Alegre VB, Oshima JEF, de Barros AE, Paviolo A, de la Torre JA, Lima F, McBride RT, Cunha de Paula R, Cullen L, Silveira L, Kantek DLZ, Ramalho EE, Maranhão L, Haberfeld M, Sana DA, Medellin RA, Carrillo E, Montalvo VH, Monroy-Vilchis O, Cruz P, Jacomo ATA, Alves GB, Cassaigne I, Thompson R, Sáenz-Bolaños C, Cruz JC, Alfaro LD, Hagnauer I, Xavier da Silva M, Vogliotti A, Moraes MFD, Miyazaki SS, Araujo GR, Cruz da Silva L, Leuzinger L, Carvalho MM, Rampim L, Sartorello L, Quigley H, Tortato FR, Hoogesteijn R, Crawshaw PG, Devlin AL, May Júnior JA, Powell GVN, Tobler MW, Carrillo-Percastegui SE, Payán E, Azevedo FCC, Concone HVB, Quiroga VA, Costa SA, Arrabal JP, Vanderhoeven E, Di Blanco YE, Lopes AMC, Ribeiro MC. 2021. Environmental and anthropogenic factors synergistically affect space use of jaguars. *Current Biology* 31:3457-3466.e4. DOI: 10.1016/j.cub.2021.06.029.

Webster MM, Rutz C. 2020. How STRANGE are your study animals? *Nature* 582:337–340. DOI: 10.1038/d41586-020-01751-5.

Wickham H. 2016. ggplot2: Elegant Graphics for Data Analysis.

Wickham H, François R, Henry L, Müller K, Davis V. 2022. dplyr: A Grammar of Data Manipulation.

Yu X, Wu NC, Ge L, Li L, Zhang Z, Lei J. 2022. Artificial shelters provide suitable thermal habitat for a cold-blooded animal. *Scientific Reports* 12:5879. DOI: 10.1038/s41598-022-09950-y.

Figure1

Click here to access/download;Figure;Fig1.tif

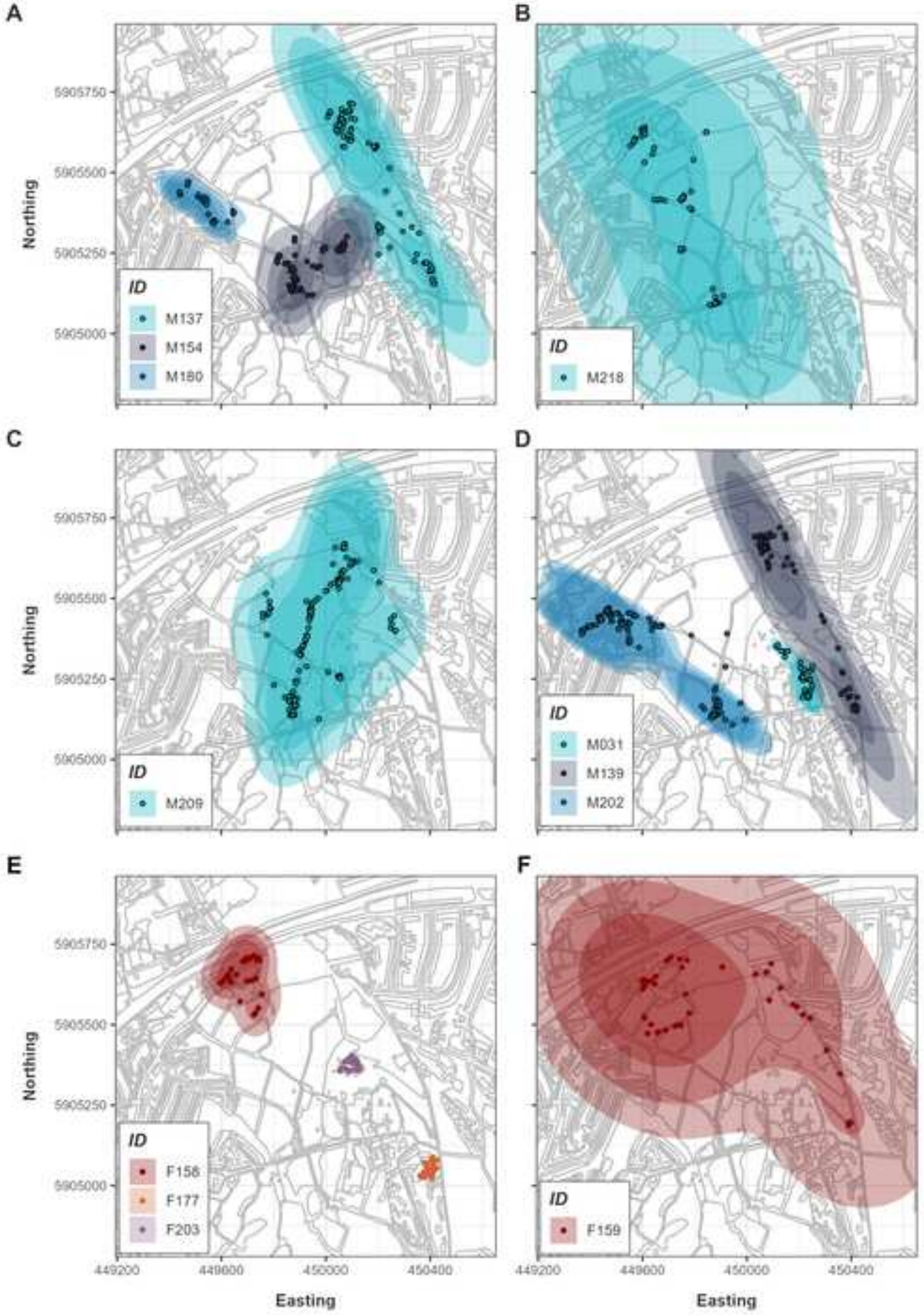

Figure2

Click here to access/download;Figure;Fig2.tif

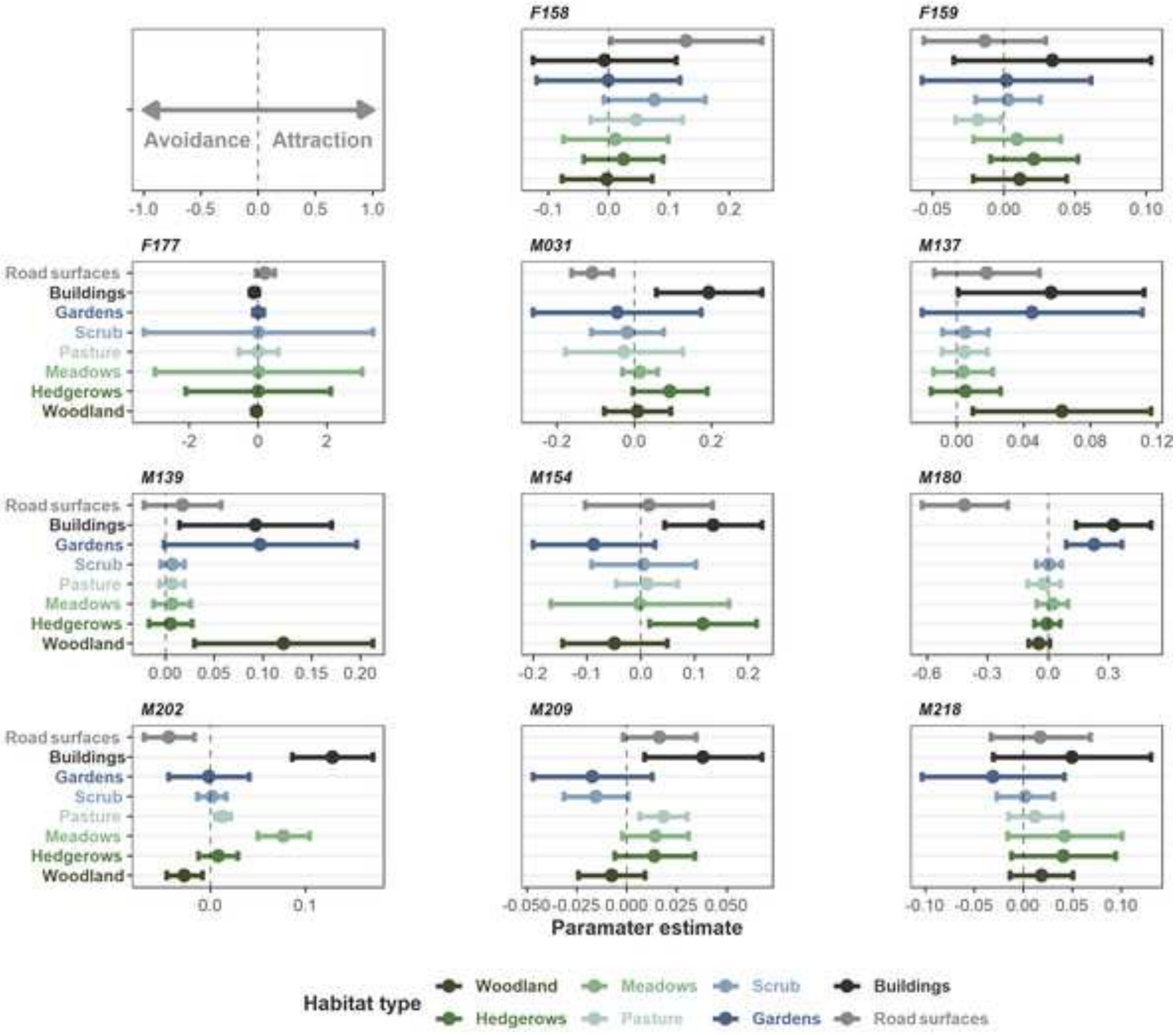

Figure3

Click here to access/download;Figure;Fig3.tif

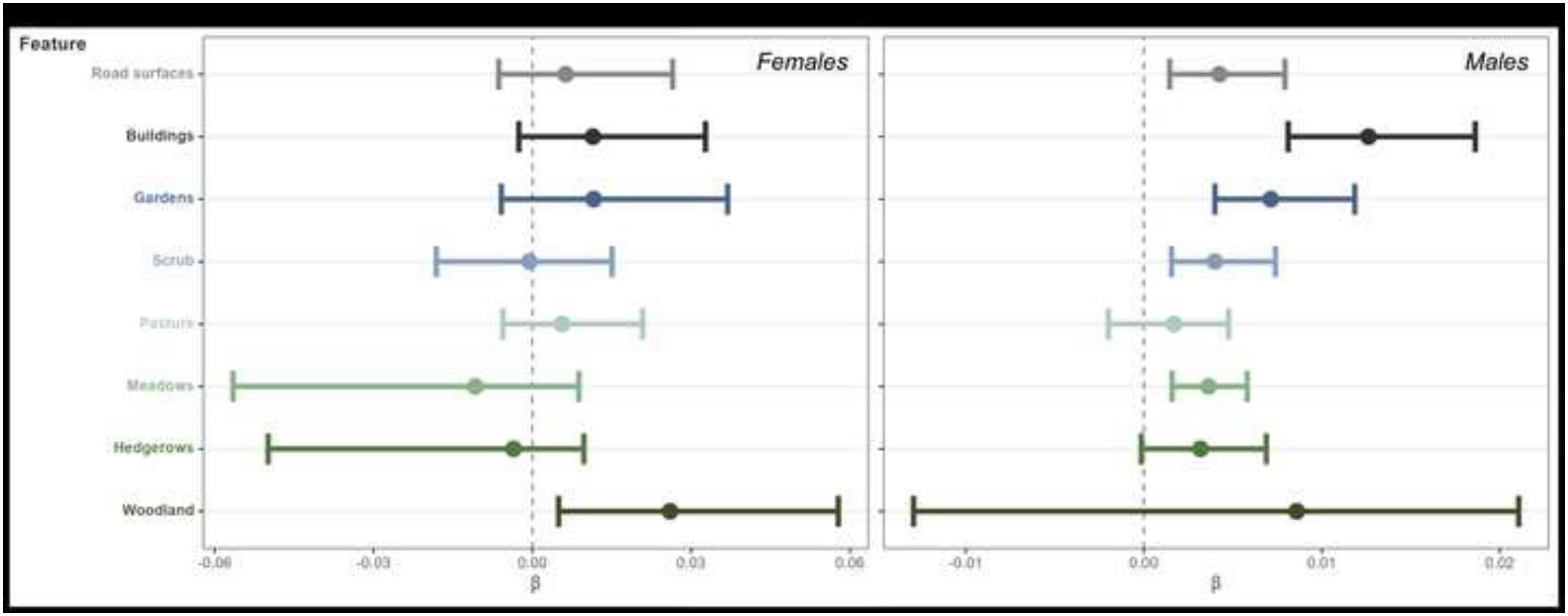

Figure4

Click here to access/download;Figure;Fig4.tif

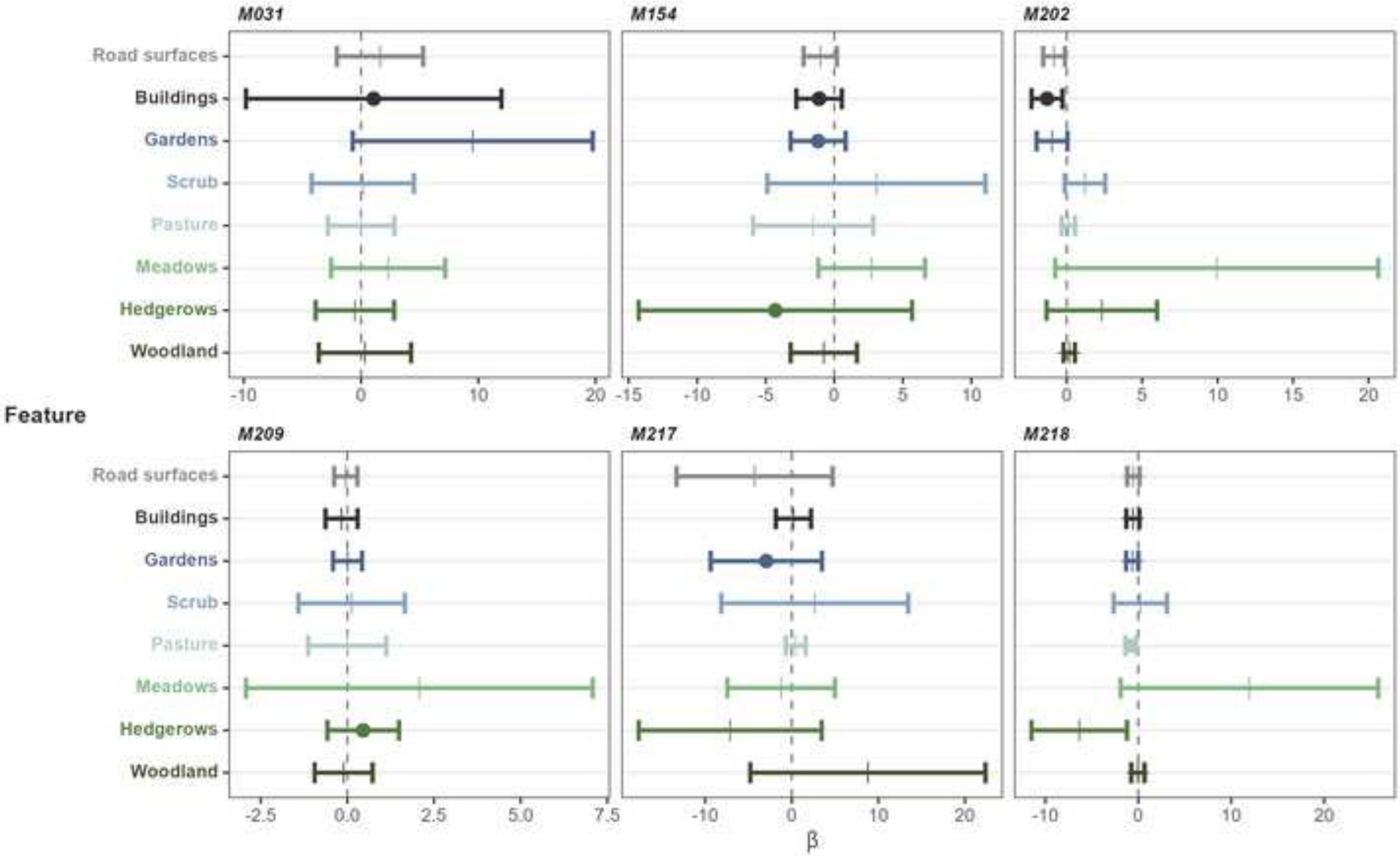

Figure5

Click here to access/download;Figure;Fig5.tif

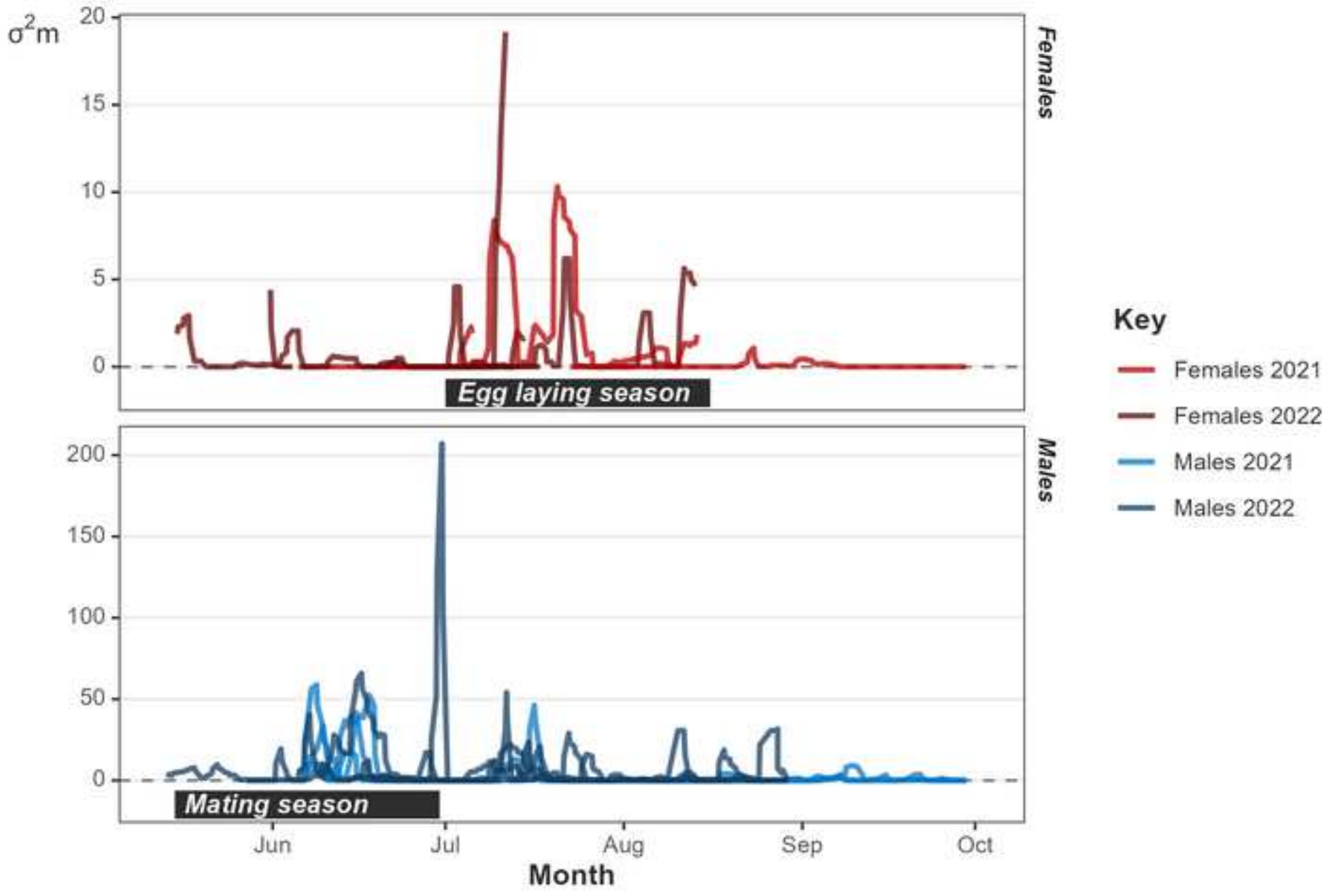

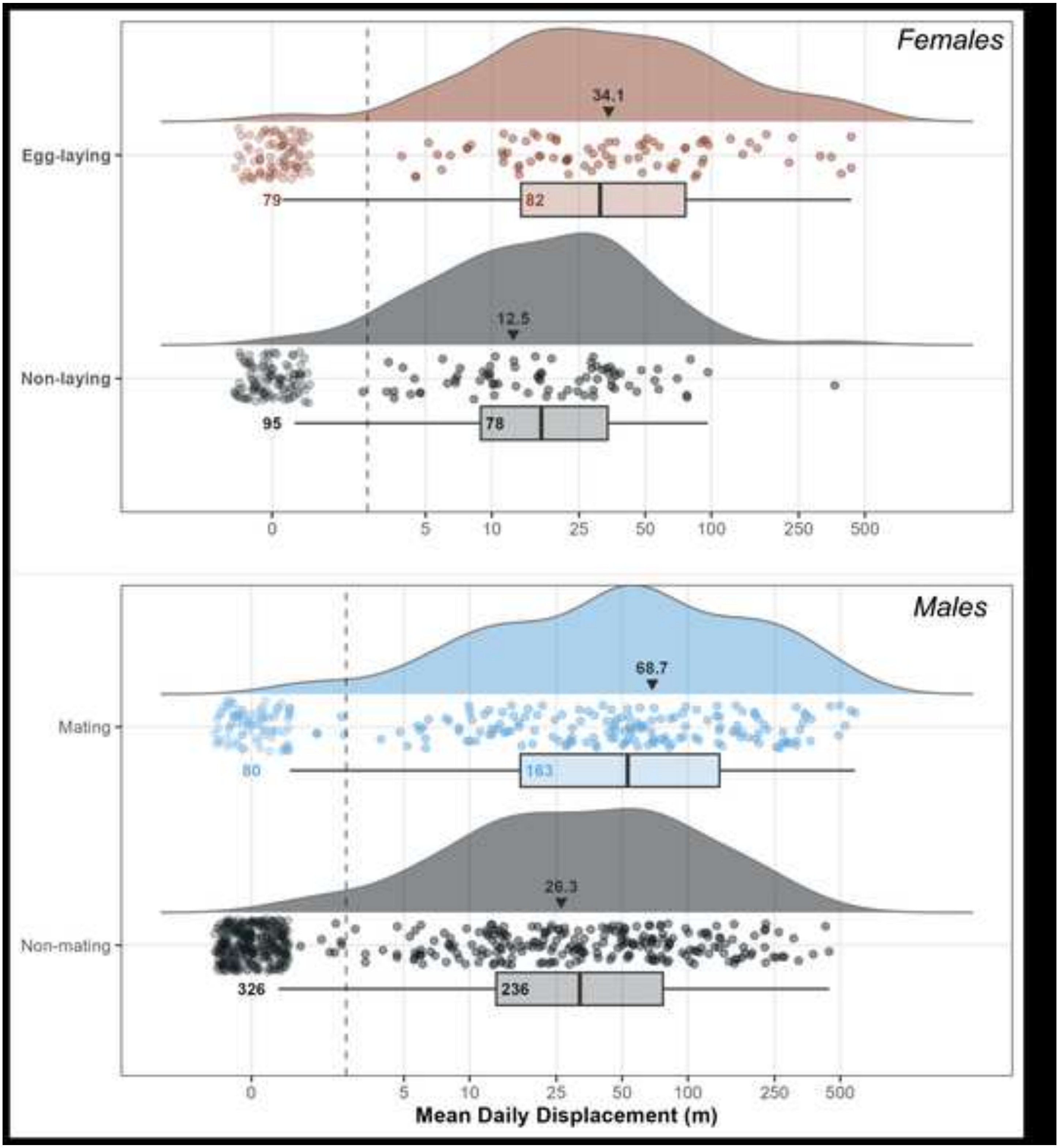
Females
34.1
Egg-laying
79
82
12.5
Non-laying
95
78
0
5
10
25
50
100
250
500
Males
68.7
Mating
80
163
26.3
Non-mating
326
236
0
5
10
25
50
100
250
500
Mean Daily Displacement (m)

Click here to access/download
Supporting Information
Supplementary material.docx
