# Supplementary material for: A reliance on human habitats is key to the success of an introduced predatory reptile
Source: PLoS One. 2025 Feb 5;20(2):e0310352. doi: 10.1371/journal.pone.0310352 (PMC11798526; doi:10.1371/journal.pone.0310352)
Supplement: Attachment — Submitted filename: Response to Reviewers.docx [file pone.0310352.s022.docx]

Response to reviewers: PONE-D-24-37502, Major et al

Thank you very much for the thorough review and consideration of our work. We have added the following elements based on editors’ comments:

General comments

- Changed corresponding author affiliation to Bangor University (1) and Bournemouth University (2) which reflects the fact that TM conducted this work during his PhD in Bangor.
- Added amended role of funder statement to cover letter.
- Added link to Figshare data and code repository, and ethics statement, in ‘Limitations and data statement’ of methods.
- Made the data and code available and no longer embargoed.
- Corrected the supporting information section of the manuscript and the separate supporting information file.

Comments to reviewers:

**Reviewer 1**

- Clarified how the snakes escaped from the Welsh mountain Zoo on line 70, “Snakes were introduced to Colwyn Bay, North Wales when an unknown number of individuals escaped from the Welsh Mountain Zoo in the 1970s due to structural failure of their enclosure.”
- Line 147, clarified Aesculapian snakes are primarily diurnal.
- Line 450, added line to discussion mentioning use of man-made structures in the northern part of the range and cited Kovar et al 2016.
- Made all minor grammatical changes suggested in the marked up PDF, both in text and references.

**Reviewer 2**

Lines 125-126: Is there a reference for this or perhaps just specify where this data comes from?

- Added explanation that the time taken to find snakes comes from our own unpublished data

Lines 127: Body diameter over what size approximately?

- Added explanation, “we radio-tracked any available adult snakes with sufficient body diameter to carry a transmitter, which was approximately 20 mm at the beginning of the posterior third of the snake excluding the tail.”

Discussion: I noticed there is no mention of Aesculapian snake prey species or what they are preying on in this introduced range? As this is a major component of driving species persistence, habitat use, and potential impacts to local fauna it is probably worth a mention somewhere in the discussion?

- Added a line at 457: “As snakes in this population prey predominantly on small mammals, supplemented with birds (unpublished data), these locations may provide foraging opportunities in addition to shelter”.

Further, given the general global focus of introduced predators/species is environmental impact it seems odd not to mention a perspective on this.

- Added a line in the conclusion, “While Aesculapian snakes are present in the fossil record of the UK, they have been absent for likely 300,000 years (Ashton et al., 1994). **Their impact on native fauna remains uncertain, but they coexist with similar assemblages elsewhere, particularly in western mainland Europe.”**
